# Uniform Expression and Relatively Small Position Effects Characterize Sister Transformants in Maize and Soybean

**DOI:** 10.3389/fpls.2019.01209

**Published:** 2019-10-24

**Authors:** Scott D. Betts, Sutirtha Basu, Joy Bolar, Russ Booth, Shujun Chang, A. Mark Cigan, Jeffry Farrell, Huirong Gao, Kristi Harkins, Anthony Kinney, Brian Lenderts, Zhongsen Li, Lu Liu, Michelle McEnany, Jasdeep Mutti, Dave Peterson, Jeffry D. Sander, Chris Scelonge, Xiaoyi Sopko, Dave Stucker, Emily Wu, N. Doane Chilcoat

**Affiliations:** ^1^Corteva Agriscience, Johnston, IA, United States; ^2^Benson Hill Biosystems, Inc. St. Louis, MO, United States; ^3^Genus PLC, DeForest, WI, United States; ^4^Kenfeng Seed, Harbin, China; ^5^KWS Gateway Research Center, LLC, St. Louis, MO, United States

**Keywords:** transformant, transgene expression, site-specific integration, position effect, insertion site

## Abstract

Development of transgenic cell lines or organisms for industrial, agricultural, or medicinal applications involves inserting DNA into the target genome in a way that achieves efficacious transgene expression without a deleterious impact on fitness. The genomic insertion site is widely recognized as an important determinant of success. However, the effect of chromosomal location on transgene expression and fitness has not been systematically investigated in plants. Here we evaluate the importance of transgene insertion site in maize and soybean using both random and site-specific transgene integration. We have compared the relative contribution of genomic location on transgene expression levels with other factors, including cis-regulatory elements, neighboring transgenes, genetic background, and zygosity. As expected, cis-regulatory elements and the presence/absence of nearby transgene neighbors can impact transgene expression. Surprisingly, we determined not only that genomic location had the least impact on transgene expression compared to the other factors that were investigated but that the majority of insertion sites recovered supported transgene expression levels that were statistically not distinguishable. All 68 genomic sites evaluated were capable of supporting high-level transgene expression, which was also consistent across generations. Furthermore, multilocation field evaluation detected no to little decrease in agronomic performance as a result of transgene insertion at the vast majority of sites we evaluated with a single construct in five maize hybrid backgrounds.

## Introduction

The development of genetically modified organisms or cell lines for commercial purposes involves inserting DNA sequences into the nuclear genome to generate a trait of interest. The product development process is typically comprised of multiple steps, including optimization of protein coding sequence, selection of cis-regulatory elements, transgene synthesis, generation of transformed plants or transformants, and selection of transformants based on molecular and phenotypic criteria. It has been observed that independent sister transformants, that is, transgenic cells/organisms generated using the same DNA construct and containing one or more DNA insertions or “events” can exhibit dramatically different transgene expression levels (Strauss and Sax, 2016). This wide variation in transgene expression among sister transformants has led to the inference that genomic insertion site plays a central role in transgene expression (Matzke and Matzke, 1998; Cocciolone et al., 2000; Cantos et al., 2014) and has led to a product development paradigm wherein large numbers of sister transformants are generated and phenotyped to select a single transgenic event that has an expression level leading to appropriate trait efficacy in elite hybrids or varieties suitable for commercialization (Sachs et al., 1998; Bradford et al., 2005; Burns et al., 2015).

Several causes of chromosomal insertion site effects, or “position effects,” on transgene expression activity have been described in mechanistic terms. Some differences in gene expression attributable to genomic location are likely caused by interaction with nearby genes, a phenomenon that has been clearly demonstrated in plants and other organisms (Eszterhas et al., 2002). For example, strong enhancers are known to increase the expression of nearby genes (Shakes et al., 2014), and upstream genes are known to repress downstream genes through a phenomenon known as transcriptional interference (Corbin and Maniatis, 1989). Both of these effects occur over relatively short distances of ≤20 kb (Akhtar et al., 2013). Position effects have also been attributed to higher order chromatin structural variation.

Large-scale investigations of the frequency and magnitude of position effect have come to highly varied conclusions, which may be attributable to different experimental methodologies or to differences between species. In mammalian cells, transgene expression was found to vary more than 1,000-fold based on genomic location (Akhtar et al., 2013). In that study, different chromosomal regions were found to be permissive or nonpermissive to expression, and chromatin structure was found to be predictive of expression level. Such findings indicate that chromosomal location is an important factor in transgene expression. In *Drosophila*, a more subtle position effect has been observed (Markstein et al., 2008), varying over about a three-fold range. Moreover, particular insertion sites were demonstrated to consistently affect the expression of multiple reporter genes. The *Saccharomyces* Genome Deletion library (Giaever et al., 2002) has enabled systematic and comprehensive analysis of position effect across multiple insertion sites in yeast. Wu et al. (2017) reported a 13-fold range in transgene expression levels among independent events at more than 1,000 insertion sites across all 16 yeast chromosomes. Other studies that looked at fewer insertion sites in yeast have reported similar dynamic ranges of transgene expression of up to about an order of magnitude (Flagfeldt et al., 2009; Chen et al., 2013).

Chromosomal location is thought to have a significant influence on transgene expression in plants. Several studies in plants have suggested that variability in transgene expression among sister transformants reflects real differences due to genomic location. An analysis of five early reports from 1985 to 1991 compared relative transgene expression among five sets of transformants in tobacco expressing six different transgenes (Peach and Velten, 1991). The authors concluded that the five sets of transformants followed the same distribution of transgene expression levels—from very high to very low in each case. These early studies predated the development of high-throughput molecular characterization of transgenic events. By contrast, studies in the last 25 years have attributed at least some of this variability in transgene expression levels to copy number variability among transformants. For example, in plants, it is well known that high-copy number transformants have variable levels of transgene expression and often exhibit transcriptional silencing, which may reflect a host response that originally evolved to silence transposons (Kumpatla and Hall, 1998; Kooter et al., 1999; De Buck et al., 2004; Schubert et al., 2004). Silencing or variable transgene expression levels have been observed; for example, one investigation concluded that approximately 30% of insertion sites experienced silencing or impaired transgene expression (Francis and Spiker, 2005), whereas another study found a high proportion of transformants to be unstable in transgene expression levels over a small number of generations (Iglesias et al., 1997). Even when silenced events and multicopy transformants are excluded, more than 10-fold variation in gene expression has been attributed to genomic location (Day et al., 2000; Chawla et al., 2006). These large differences in gene expression imply that genomic location is an important component of transgene activity. However, in many of these cases and in particular prior to the development of high-throughput molecular characterization and DNA sequencing, confounding effects, such as the intactness and copy number of transgenic insertions, were not routinely confirmed. Because of results like these, the concept of “genomic safe harbors”—genomic locations that are capable of supporting appropriate and consistent levels of expression for any transgene of interest—has been introduced in both plants and mammalian systems (Cantos et al., 2014; Wallen et al., 2015).

With the use of the FLP/FRT recombinase system and deep sequencing-enabled molecular characterization technologies, we have been able to systematically evaluate position effect on transgene expression in the important crop plants corn and soybean. In contrast to the studies cited above, we have found that the vast majority of genomic locations tested support similar levels of transgene expression at an early vegetative stage, without fitness impairment, when including only those transformants with a single, intact transgene insertion verified at the nucleotide level. Our results suggest that the significance of genomic position effect on transgene expression in crop plants, at least based on gene expression analysis at an early developmental stage of the transgenic plant in leaf and root, is less than previously thought. These findings present a paradigm shift for transformation-based commercial product development.

## Results

### Production and Molecular Characterization of Transgenic Insertion Events in Maize and Soybean

A series of transformation experiments was initiated in corn and soybean with the goal to generate multiple independent sets of sister transformants containing identical transgenic sequences at different genomic locations. Sister transformants are defined here as transgenic plants or lines that contain independent but otherwise identical transgene insertions/events. Transgenic DNA was introduced into maize cells either by *Agrobacterium* for insertion at random genomic locations or by particle bombardment (biolistic transformation) for site-specific integration (SSI). In soybean, DNA delivery was by both particle bombardment and *Agrobacterium*.

Transformants frequently do not contain a single-copy intact insertion of the transgenic DNA and therefore, not surprisingly, have highly variable transgene expression. This is true for transformation methods using either biolistic or *Agrobacterium*-mediated DNA delivery (e.g., Kohli et al., 2003). Using a combination of quantitative PCR (qPCR) and Southern-by-Sequencing (SbS) (Zastrow-Hayes et al., 2015), we evaluated transformants generated by *Agrobacterium* transformation and by SSI and identified individual events that contained only the intended DNA insertion. qPCR was used to determine the copy number of transgenic sequences, whereas SbS used a combination of oligo-based target enrichment and Illumina-based sequencing to determine the presence and location of transgenic DNA. For random transgene insertions, we used qPCR-based assays, targeting each of the genes in the construct to select for single-copy transformants that were also negative for transformation “helper” genes and *Agrobacterium* backbone DNA. These single-copy helper-free backbone-negative transformants were subsequently analyzed by SbS, and only events with fully intact insertions were included in the analyses of transgene expression. For SSI transformants, we conducted qPCR to confirm the presence of correct junction sequences and to confirm the absence of helper/*Agrobacterium* backbone DNA. All SSI transformants were subsequently analyzed by SbS for intactness of the transgenic event, as described above for random insertion transformants. Transformants with imperfect DNA insertions were eliminated from the transgene expression analyses (see example in **Figure S1**).

### Effect of Chromosomal Position on Gene Expression From Single-Copy T-DNA Insertions

**Figure 1:**
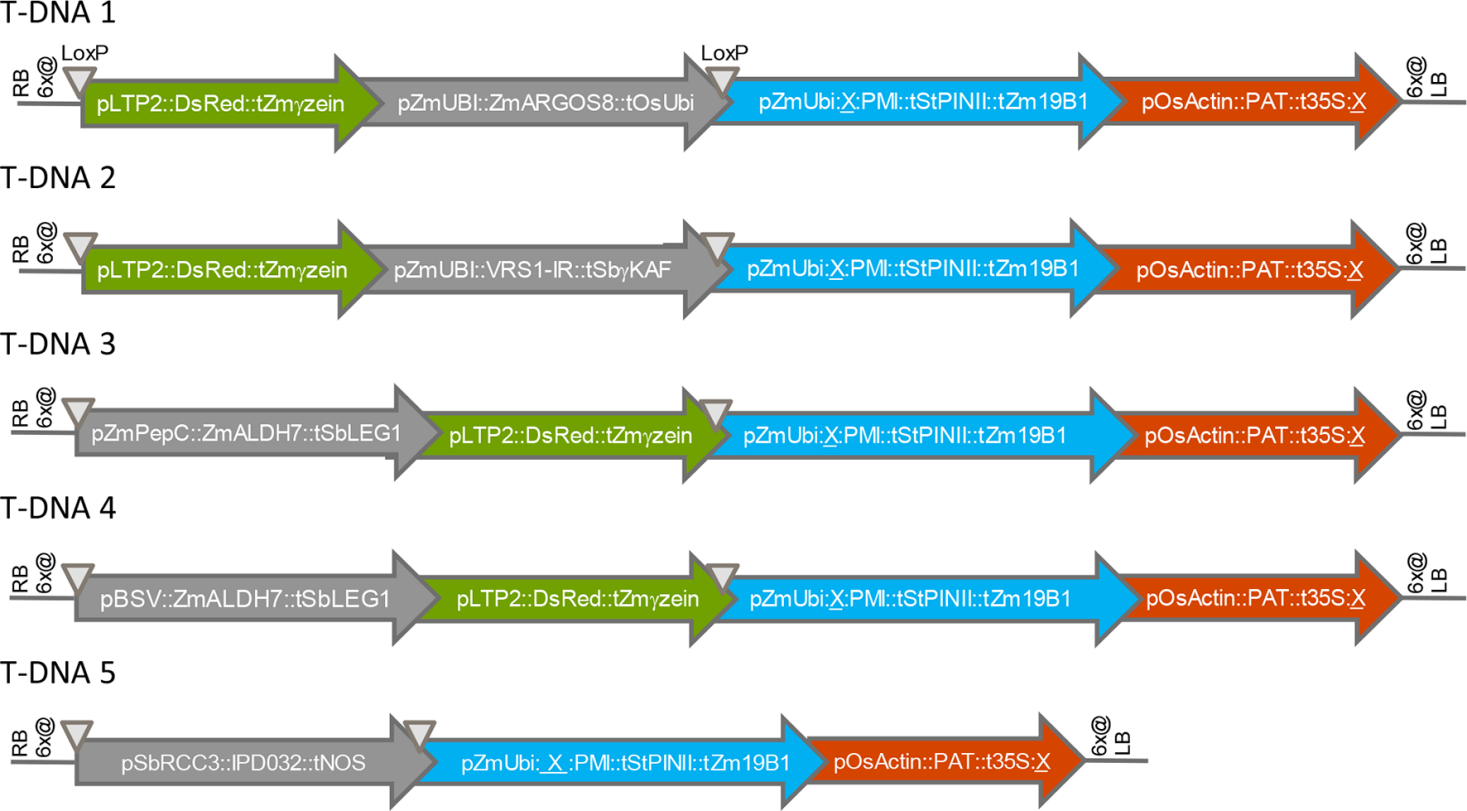
Structures of T-DNA constructs introduced into maize by *Agrobacterium* transformation. RB, right T-DNA border; LB, left T-DNA border; 6x@, stop codons in all six reading frames; p, promoter element, t, terminator element; ∇, LoxP site; X, FRT site. Arrows indicate the direction of transcription.

We first assessed the effect of chromosomal location on transgene expression from various T-DNA insertions in maize. All transgenes were designed for overexpression of the encoded polypeptide, except for VRS1, which was expressed as hairpin RNA for silencing of an endogenous gene. **Figure 1** shows the structures of five T-DNA constructs (T-DNA 1 to T-DNA 5) containing 3-4 transgene expression cassettes (“molecular stacks”) that were used to generate multiple independent sets of sister transformants in the maize inbred HC69. All five T-DNA constructs contain one PMI cassette, one PAT cassette, and one gene of interest or “trait” cassette (ARGOS8, VRS1, ALDH7, IPD032). Four of these five T-DNA constructs contain a fourth cassette encoding a fluorescent color marker protein. Detailed names and source organisms of all DNA components used in expression cassettes are provided in **Table S1**.

**Figure 2:**
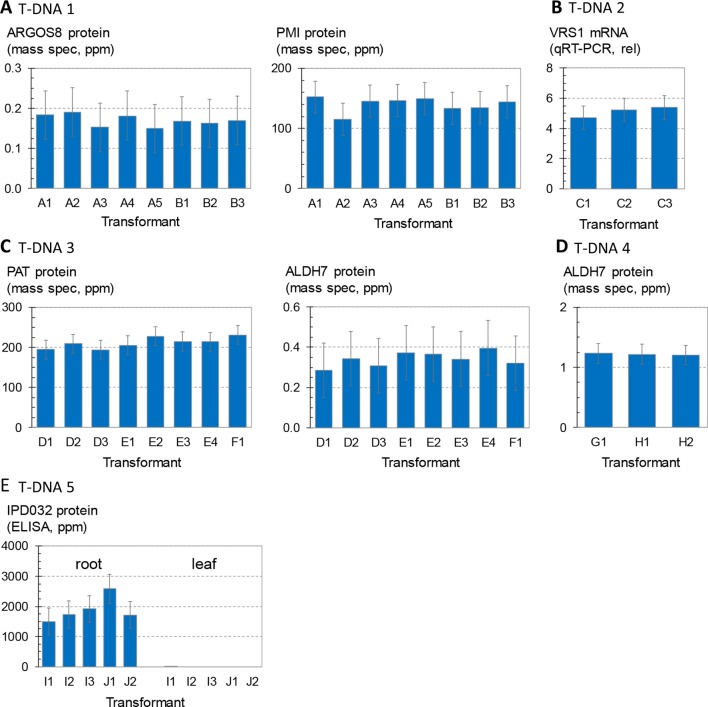
Analysis of transgene expression levels from T-DNA insertions in maize. Transformant names (x axis) indicate independent transformation experiments (large case letter) and independent insertion events from the same transformation experiment (number). For example, the data in **(A)** are from five independent transformants or events from experiment A and three independent transformants or events from experiment B. **(A)** and three independent transformants or events from experiment B. Each bar is the average of four replicates of three to four plants each, and error bars indicate 95% confidence intervals. Results from 447 plants are summarized. **(A)** Concentrations of ARGOS8 and PMI in leaf extracts of plants containing T-DNA 1. Each bar is the mean of 14–16 plants. **(B)** Concentrations of VRS1 transcripts in leaf extracts of plants containing T-DNA 2. Each bar is the mean of 15–16 plants. **(C)** Concentrations of PAT and ALDH7 in leaf extracts of plants containing T-DNA 3. Each bar is the mean of 15–16 plants. **(D)** Concentrations of ALDH7 in leaf extracts of plants containing T-DNA 4. Each bar is the mean of 15–16 plants. **(E)** Concentrations of IPD032 in root and leaf extracts of plants containing T-DNA 5. Each bar is the mean of 14–18 plants. For events containing the IPD032 transgene, each replicate consisted of two to five plants hemizygous for the IPD032 transgene.

**Figure 2** shows the variation in transgene expression among multiple sets of sister transformants generated with the five molecular stack vectors using random integration technology. Regenerated (T0) plants containing a single intact copy of one of the T-DNA constructs in **Figure 1** were crossed to nontransgenic recurrent parent (HC69) to generate F1 generation progeny. The F1 progeny are expected to be 50% hemizygous for the transgenic insertion. Transgenic seeds were sorted by visual detection of the red fluourescent color marker (**Figure 1**), and null segregants were discarded. These transgenic F1 seeds were planted in the greenhouse in a randomized complete block design, and leaf and root tissue were sampled for transgene expression analysis as noted in the figure legend.

**Figure 2A** compares expression levels of the maize ARGOS8 transgene, measured as ARGOS8 protein concentration, for a set of eight sister transformants containing T-DNA 1. The mass spectrometry method used here measures recombinant protein concentrations above any background signal attributable to the corresponding endogenous protein (Schacherer et al., 2017). Statistical testing for differences in means across the eight transformants showed no significant differences. **Figure 2A** also shows PMI protein concentrations measured in the same leaf extracts. PMI protein concentrations were also consistent, and no significant differences in the means were found among the eight transformants either. **Figure 2B** compares three sister transformants containing the T-DNA 2 insertion, which has the same molecular stack design as T-DNA 1, except for the gene of interest. The gene of interest in T-DNA 2 contains the maize Ubi promoter and first intron driving an inverted repeat of a fragment of the maize VRS1 gene. Transcript levels from this VRS1 inverted repeat in leaf extracts were consistent, with no significant differences in mean transcript levels across the three sister transformants. **Figure 2C** shows the concentrations of PAT and ALDH7 proteins in leaf extracts of eight sister transformants from three transformation experiments introducing T-DNA 3. The mean concentrations across all eight events were not significantly different for either protein. **Figure 2D** summarizes ALDH7 protein concentrations in leaf extracts from three transformants with the strong, constitutive viral promoter BSV driving transcription of the ALDH7 CDS (T-DNA 4). The concentrations of ALDH7 protein in these three events from two transformation experiments were almost identical. The chart in **Figure 2E** shows IPD032 protein concentrations in leaf and root extracts from young maize plants transformed with T-DNA 5. The IPD032 coding sequence is under the transcriptional control of the root-specific promoter RCC3. The IPD032 protein was not detected in leaf extracts, as expected for a root-specific promoter. The mean concentration of IPD032 in root extracts ranged from about 1,500 to 2,500 ppm across the five sister transformant lines (CV 22%). IPD032 concentrations in root extracts were not significantly different (95% CI) between four of the five events, but events I1 and J1 were significantly different from each other by a factor of about 1.6X (CI 1.05–3.0).

In summary, among the 27 single-copy, intact transgenic insertions analyzed, the expression levels of six different transgenes in five molecular stack constructs (each tested at 3–8 genomic insertion sites) were highly consistent and reproducible within and across sister transformants.

### Effect of Chromosomal Position on Gene Expression From SSI Events

In the previous experiments, we observed no statistically significant differences between five sets of sister transformants with one exception (**Figure 2E**, transformants I1 and J1), despite using highly replicated experimental designs and robust assays. However, we did observe some transformants with numerically higher or lower levels of transgene expression. This variation could come from many origins, certainly including chromosomal location, but also including somaclonal effects or experimental noise (e.g., greenhouse heterogeneity, assay accuracy). To understand if these minor differences are related to genomic insertion site, we analyzed sister transformants with identical chromosomal insertion sites. This was accomplished using site-specific insertion (SSI) technology.

In these experiments, primary transformants were generated by random *Agrobacterium* or biolistic transformation. In subsequent steps, secondary SSI transformants were generated either directly in the next step (soybean) or following an intermediate Cre-mediated excision step to remove trait genes from the primary transgenic event (maize). Additional details describing the generation of transformants containing SSI landing sites are provided in **Figures S2 and S3**.

**Figure 3:**
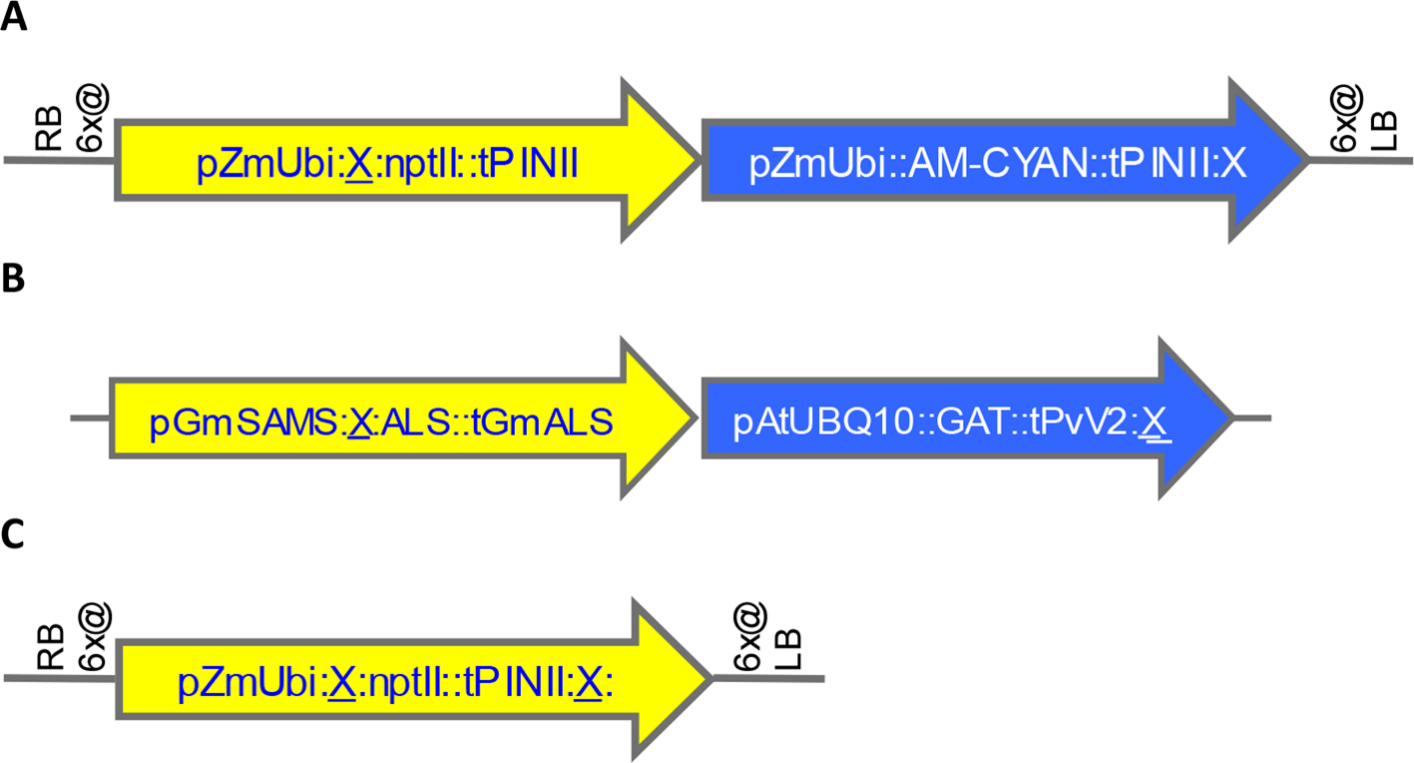
Structures of landing sites and transformants generated by random transformation, site-specific integration (SSI), and homology-directed repair (HDR). X, FRT site. **(A)**. Maize landing site structure generated by SSI from preexisting random transformants. See structure of example precursor transformant in **Figure S3A**. **(B)**. Soybean transformant structure generated by SSI from preexisting random events shown in **Figure S3B-C**. **(C)**. Maize SSI site structure generated using CRISPR-Cas9 technology.

**Figure 4:**
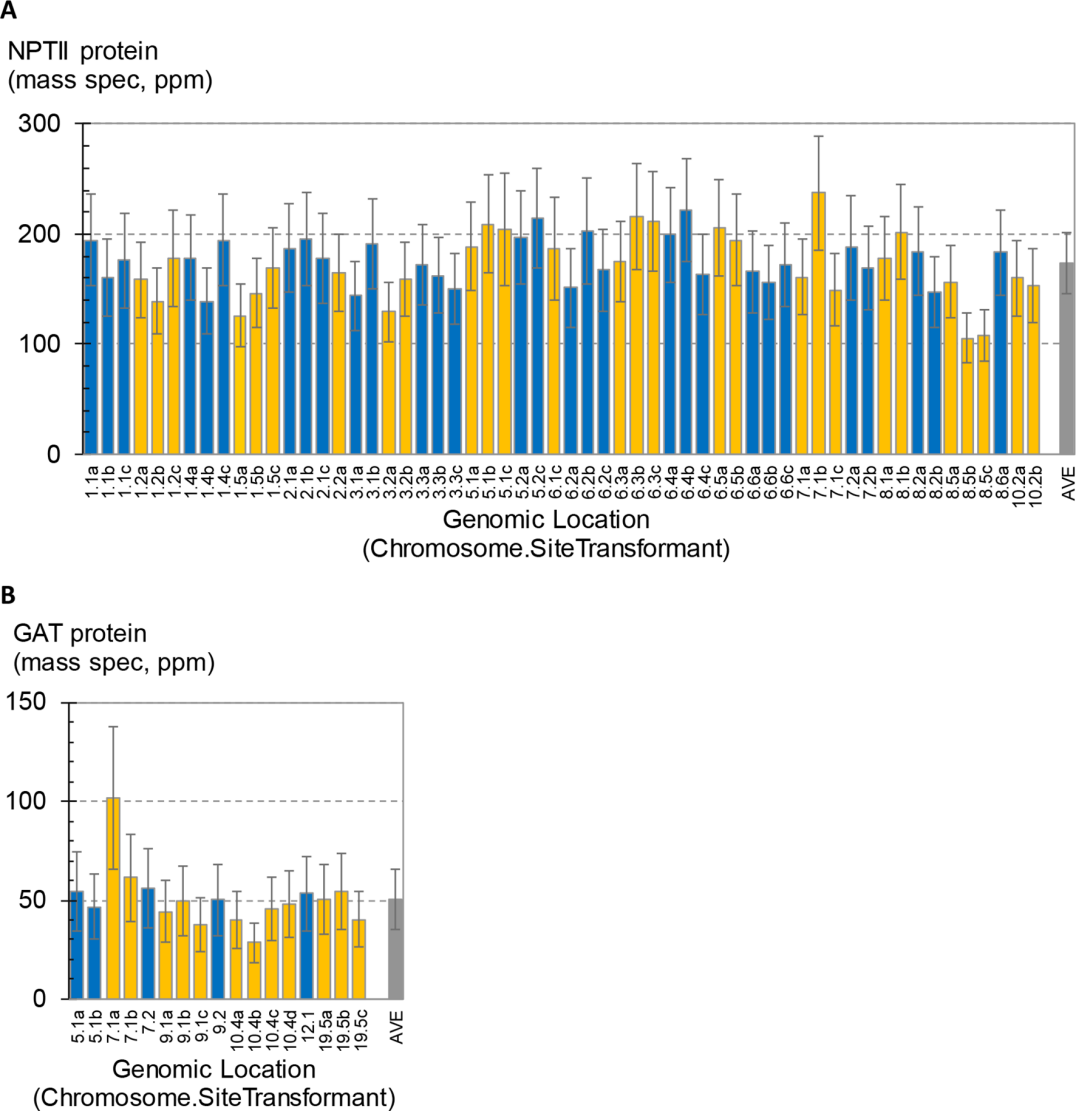
Measurement of transgene expression levels in transformants generated by site-specific integration (SSI). One to four independent SSI events were characterized at each genomic insertion site. The x-axis labels indicate the chromosome number followed by site number (arbitrary) and ending with a small case letter to identify independent events at the same site (a–d). Error bars on blue and gold bars indicate 95% confidence intervals. Gray bars indicate average ± standard deviation of all events. **(A)** Concentrations of NPTII protein in leaf extracts from 58 independent maize transformants were measured by mass spectrometry. Results from 840 plants are summarized; each bar is the mean of 10–16 plants. **(B)** Concentrations of GAT protein in leaf extracts from 17 independent soybean SSI transformants. Results from 167 plants are summarized; each bar is the mean of 9–10 plants.

A total of 58 SSI transformants in maize were confirmed to have the SSI landing site structure shown in **Figure 3A**. Only fully intact single-copy insertions based on SbS analysis were evaluated. F1 generation seeds hemizygous for the transgene locus were selected based on visual detection of the fluorescent color marker. These F1 seeds were then planted in flats using the standard experimental design for transgene expression analysis. The concentration of NPTII protein in leaf extracts was measured by mass spectrometry. In total, 840 maize plants were analyzed representing 58 independent SSI transformants at 24 different genomic sites. NPTII concentrations in leaf extracts ranged from 105 ppm (transformant 8.5b) to 237 ppm (transformant 7.1b) across the 58 transformants covering eight maize chromosomes (**Figure 4**). The average NPTII concentration in these 58 transformants was 174 ± 27 ppm (CV 16%).

As observed in the above analysis of primary sister transformants in maize (**Figure 2**), statistically significant differences in transgene expression levels were also observed between some of the SSI transformants in this larger set of transgenic events with identical insertion structures (**Figure 4A**). For example, transformants 8.5b and 8.5c were the lowest expressing lines in the study and were significantly lower than line 7.1b or 8.6a. However, a third independent transformant at site 8.5 (line 8.5a) accumulated NPTII protein to an intermediate level and was not significantly different than either of its molecularly identical sister transformants 8.5b and 8.5c or the higher expressing lines 7.1b and 8.6a. Similarly, transformant line 7.1c was significantly lower than its identical sister transformant 7.1b. Because the variation observed between sets of sister transformants with the same construct at different genomic locations was not greater than the variation between sister transformants with the same construct at the same location (made by SSI), we conclude that most variations in transgene expression level are not attributable to position effect/chromosomal insertion site. The observed variation may be attributable to somaclonal variation or uncontrolled experimental noise.

The effect of genomic insertion site on transgene expression was also characterized in soybean using a set of SSI events. In this experiment, a set of 17 independent SSI transformants at eight landing sites was analyzed. These transformants have the event structure shown in **Figure 3B**) (Li et al., 2010). Regenerated T0 plants were selfed to produce T1 generation seeds, and T1 generation plants were selfed to produce T2 seeds homozygous for the SSI event. T2 soybean seeds were grown in the greenhouse in a randomized complete block design with 10 plants per entry. Leaf samples were collected at the V5 stage, and the concentration of GAT protein was measured in the leaf extracts. The results for individual soybean SSI lines are shown in **Figure 4B**. Mean concentrations of GAT protein ranged from 29 ppm (transformant 10.4a) to 102 ppm (transformant 7.1a) with an average of 51 ± 15 ppm (CV 30%) across the 17 transformants. Transformant 7.1a was significantly different from five of the other 16 transformants, none of which were significantly different from each other and which included event 7.1b, a molecularly identical event at the same SSI site as 7.1a. As with SSI events in maize, most variations in transgene expression among sister SSI transformants in soybean are not attributable to chromosomal location.

### Analysis of Transgene Expression Across Generations

**Figure 5:**
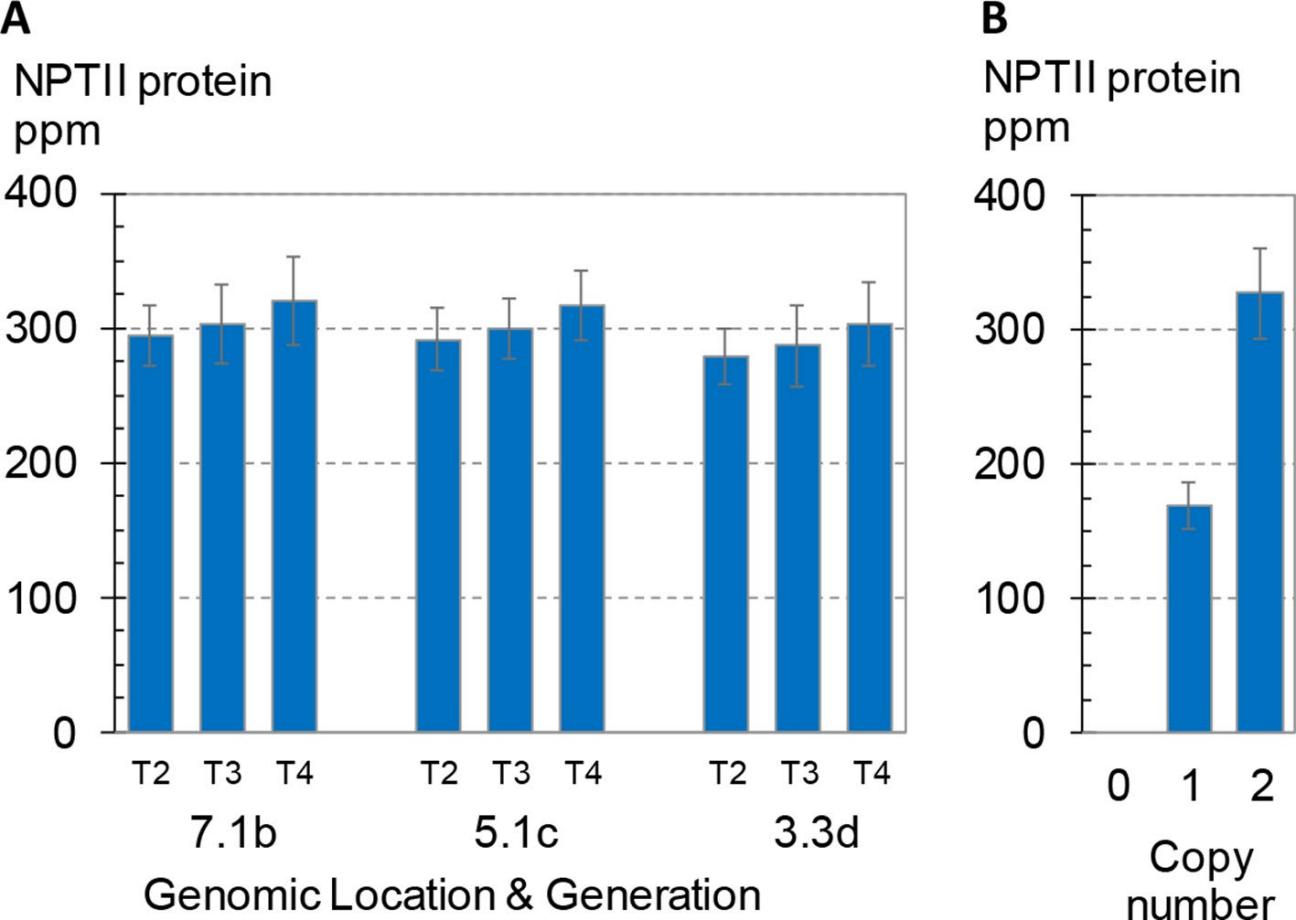
Transgene expression across three generations and its dependence on zygosity. NPTII protein concentrations of leaf extracts were measured using mass spec. Error bars indicate 95% confidence intervals. **(A)** Three generations of three independent transformants in maize. Seed for generations T2-T4, all homozygous for the transgenic event, were produced in the same field, and seeds from multiple ears were bulked. To measure expression, plants were grown in the greenhouse according to the standard design used here for expression studies except that the design comprised eight plants per rep for a total of 32 seed planted for each event-generation. Events are labeled as in **Figure 4A**. All three events were generated by SSI and have the structure shown in **Figure 3A**. **(B)** BC1F2 generation seed segregating a transgenic CRISPR-Cas targeted event in maize with the structure shown in **Figure 3C**. Kernels were planted according to the standard design for leaf expression analysis but with an increased number of both replicates (12) and plants per replicate (8). Transgene zygosity (null, hemizygous, homozygous) was determined by qPCR from leaf punches from each plant in the study. Overall, the 12 replicates each contained zero to five homozygous plants and one to six hemizygous plants. Each bar is the mean of 20–21 total plants.

In addition to position effect *per se*, various epigenetic and genetic factors such as transcriptional and posttranscriptional gene silencing and transgene dosage have been implicated in variation in transgene expression (Lakshmanan et al., 2005; McGinnis et al., 2006; Arteaga-Vazquez and Chandler, 2010; Heard and Martienssen, 2014). The analyses described above demonstrate consistent transgene expression in leaf and root tissues from dozens of single-copy intact insertions in maize and soybean plants; however, these studies were limited to early generations of transgenic lines (F1 in maize, T2 in soybean) and a single developmental stage (V3 in maize, V5 in soybean). As previously noted, the maize plants characterized above were all hemizygous for the transgenes of interest, whereas the soybean plants were homozygous for the GAT transgene. To evaluate generational variation in transgene expression, we measured expression levels in three consecutive generations of maize homozygous for a transgenic construct. The results are summarized in **Figure 5**. Seeds were harvested from three successive self-pollinated generations (T2–T4) of field-grown plants comprising three independent lines each homozygous for a unique SSI landing site. Bulked seeds were planted in a randomized and replicated greenhouse expression study following the standard protocol. Leaf samples were collected at about 3 weeks after planting, and NPTII protein concentrations in the extracts were measured by mass spectrometry.

As shown in **Figure 5A**, no significant differences in NPTII protein concentrations were observed across the three generations homozygous for one of three transgenic insertions. The average NPTII protein concentration for the nine means (3 sites x 3 generations) was 299 ± 13 ppm (CV 4.4%). A fourth transformant line segregating a transgenic insertion with the same nptII cassette was included in the study as a reference. This transformant was created by direct insertion of the SSI landing site using CRISPR-Cas technology (Cigan et al., 2016) and has the insertion structure shown in **Figure 3C**. **Figure 5B** shows the mean NPTII protein concentrations of hemizygous and homozygous segregants for this CRISPR-Cas9-derived SSI line. Mean concentrations of NPTII protein in leaf extracts measured by mass spectrometry were 169 and 327 ppm in nptII-hemizygous and -homozygous plants, respectively, demonstrating linear dependence of NPTII protein concentration on transgene dosage. Taken together, these results demonstrate consistent, dose-dependent expression of NPTII across three generations of three transgenic lines homozygous for the transgene.

### Promoter Choice in Construct Design Is a Primary Determinant of Transgene Expression

**Figure 6:**
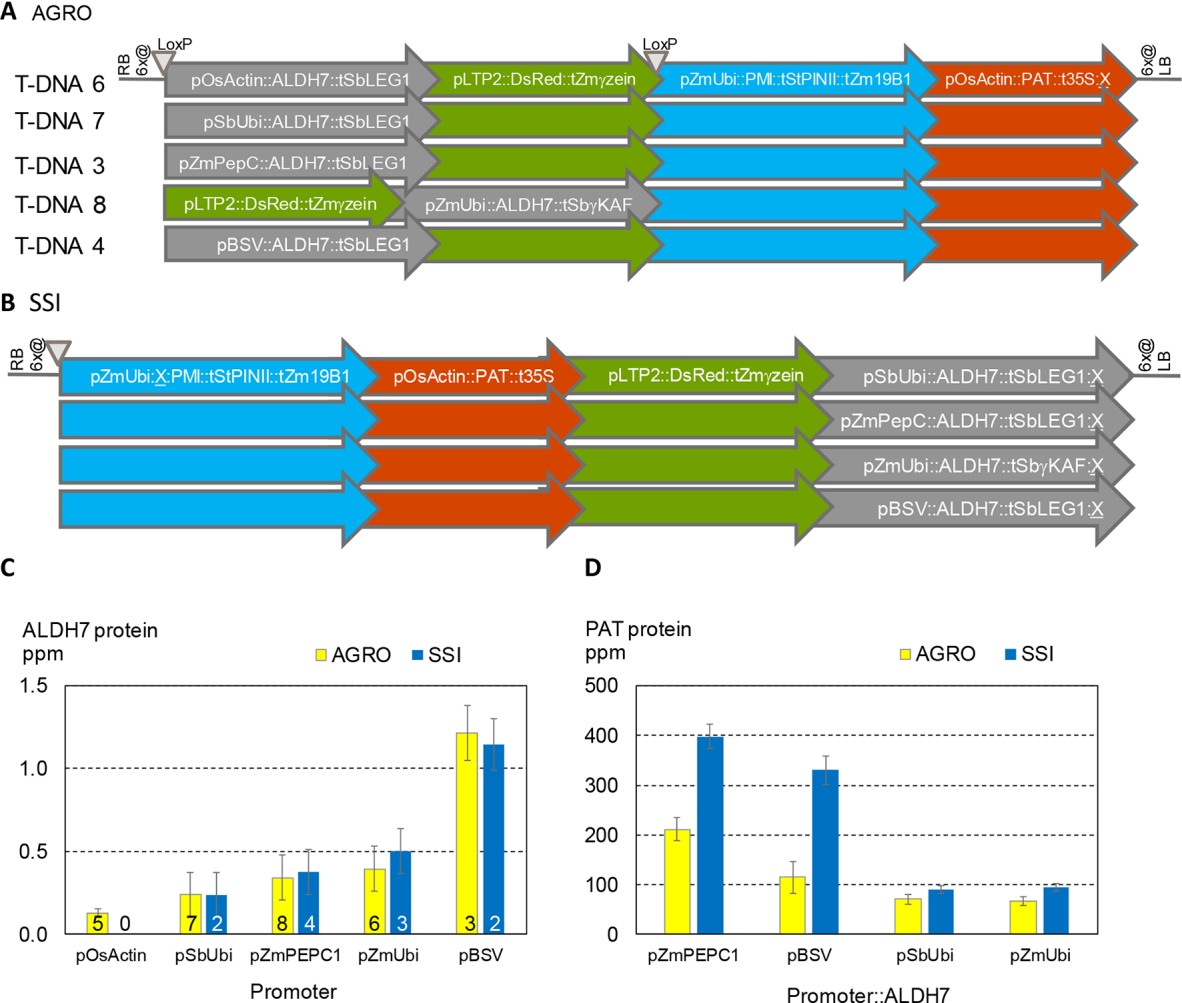
Both the choice of promoters in transgene cassettes and the order of cassettes in molecular stacks influence transgene expression levels. **(A)**. T-DNA structures in vectors used for *Agrobacterium* random (AGRO) insertion transformation. Only the transgene cassettes in gray shaded arrows vary in structure among the different vectors. **(B)**. Four site-specific integration (SSI) event structures at a single chromosome 6 site. **(C)**. ALDH7 protein concentration in young leaf extracts from random (yellow) and SSI (blue) transformants as a function of the promoter driving transcription of the ALDH7 gene. Numbers on bars indicate the number of independent transformant lines from each construct in **(A** and **B)** that were characterized in this experiment. For example, the yellow bars for PEPC (T-DNA 3) and BSV (T-DNA 4) are means of the individual transformant values shown in **Figures 2C, D** and represent a total of 127 plants (**Figure 2C**) and 47 plants (**Figure 2D**). **(D)**. The same plants characterized in **(C)** were analyzed for PAT protein concentration, which is plotted as a function of the promoter driving ALDH7 transcription.

It is well known that different promoters can lead to different levels of gene expression. For example, ALDH protein levels are ∼3X higher when driven by the BSV promoter compared to the PEPC promoter (**Figures 2C, D**). It is also well known that nearby genes can affect one another’s expression. To better characterize the significance of promoter choice and the effect of neighboring transgenes, we studied transformants made using five related DNA constructs (**Figure 6A**). All these constructs contain the same three expression cassettes: PAT, PMI, and a color marker. All contain the ALDH7 gene. The constructs vary in the order of the cassettes and the promoter driving ALDH7. A related set of SSI molecular stack constructs with the same four cassette designs but in a different order is shown in **Figure 6B**. These SSI constructs were inserted at a single SSI landing site on chromosome 6, thus eliminating position effect as a confounding factor in expression analysis.

We analyzed the expression of ALDH7 and PAT in independent transformants containing the five T-DNA structures and four SSI structures. ALDH7 expression was significantly affected by its promoter for both *Agrobacterium* random and SSI events (**Figure 6C**). ALDH7 protein concentrations for *Agrobacterium* transformants (three to eight genomic sites) and SSI transformants (one genomic site) were not significantly different for a given promoter. Moreover, the variability (standard error) among independent transformants was similar between sets of random *Agrobacterium* transformants at different sites and sets of SSI transformants at the same insertion site.

ALDH7 expression levels were attributable primarily to the promoter driving ALDH7 and not to the relative position of the ALDH7 cassette within the transgenic construct. By contrast, PAT protein concentrations were highly influenced by the location of the PAT cassette within the random and targeted molecular stacks (**Figure 6D**). The concentration of PAT protein in leaf extracts was significantly higher for all SSI constructs compared to the corresponding T-DNA constructs. Moreover, when the promoter in the ALDH7 cassette was the maize PEPC promoter or the viral BSV promoter, PAT protein levels were about 2X and 3X higher, respectively, compared to the corresponding *Agrobacterium* constructs. These results illustrate that construct design is an important determinant of transgene expression, including both the promoter driving the transgene of interest and seemingly minor details, such as the promoter driving a transgene separated from the gene of interest by two expression cassettes. In contrast, we could detect no impact from genomic location for these promoters tested, even comparing multiple molecularly identical transformants generated through SSI with similar transformants randomly integrated throughout the maize genome.

**Figure 7:**
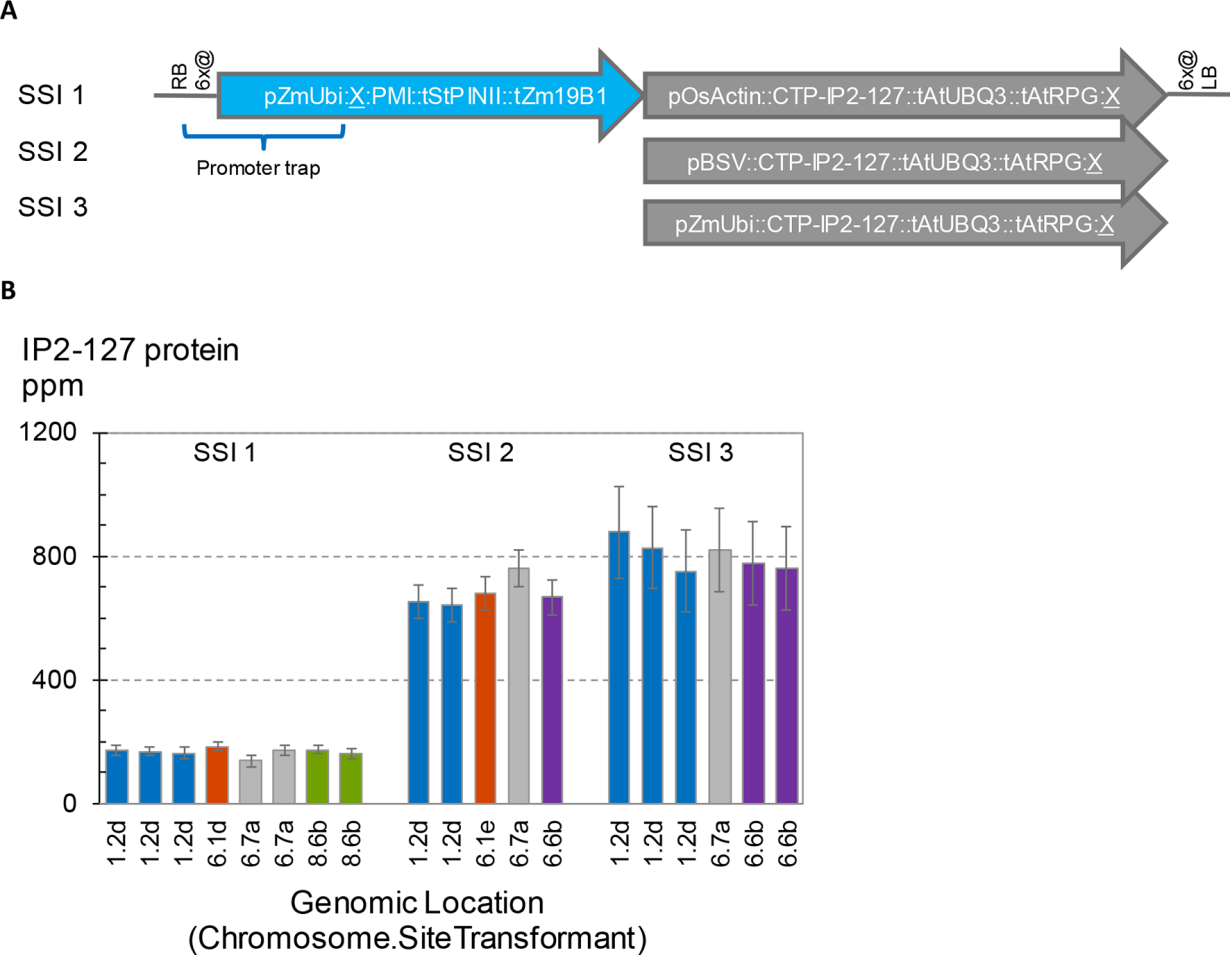
Effects of promoter choice and genomic location on transgene expression level. Three transgenes encoding IP2-127 were inserted at five site-specific integration (SSI) landing sites. A set of perfect SSI transformants were recovered and analyzed for IP2-127 protein concentration in young leaf tissue according to the standard protocol. **(A)**. Structure of SSI transformants encoding PMI and a chloroplast targeted IP2-127 protein. The PMI cassette was present in all three SSI structures. **(B)**. IP2-127 protein concentration. Genomic location indicates chromosome number followed by SSI site number. The small case letter identifies a unique SSI landing site transformant. Bars with the same color identify independent excision-exchange transformants at the same SSI landing site (molecularly identical sister transformants). The chart contains data from 365 plants; each bar is the mean of 10-20 plants.

To further investigate potential position effects on transgene expression, we initiated a series of transformation experiments with the goal to introduce three related vectors at a number of different SSI landing sites. The transgenic SSI insertions generated in these experiments are shown in **Figure 7A**. All three structures encode PMI and a chloroplast-targeted Cry2 protein designated IP2-127. The only differences between the three constructs are the promoters driving transcription of IP2-127. SSI transformants with the expected insertions were recovered from all three constructs at two landing sites (1.2, 6.7), and additional transformants from at least two of the three constructs were recovered at two additional sites (6.1, 6.6). The concentrations of IP2-127 were highly consistent across insertion sites with the same construct design (same promoter driving IP2-127) (**Figure 7B**). The coefficients of variation were 6-8% for each set of transformants with the same design. Although significant differences in expression levels were observed between a few of the transformants with the same structure (e.g., SSI I1 events at site 6.1d and one of the two identical events at site 6.7a), these differences were small compared to the differences attributable to the promoter driving IP2-127 expression. The BSV and ZmUbi1 promoters consistently supported IP2-127 protein accumulation to much higher levels than the OsActin promoter, consistent with the results above for the same promoters in the ALDH7 cassettes.

### Genetic Background Affects Transgene Expression Significantly and Independent of Insertion Site

**Figure 8:**
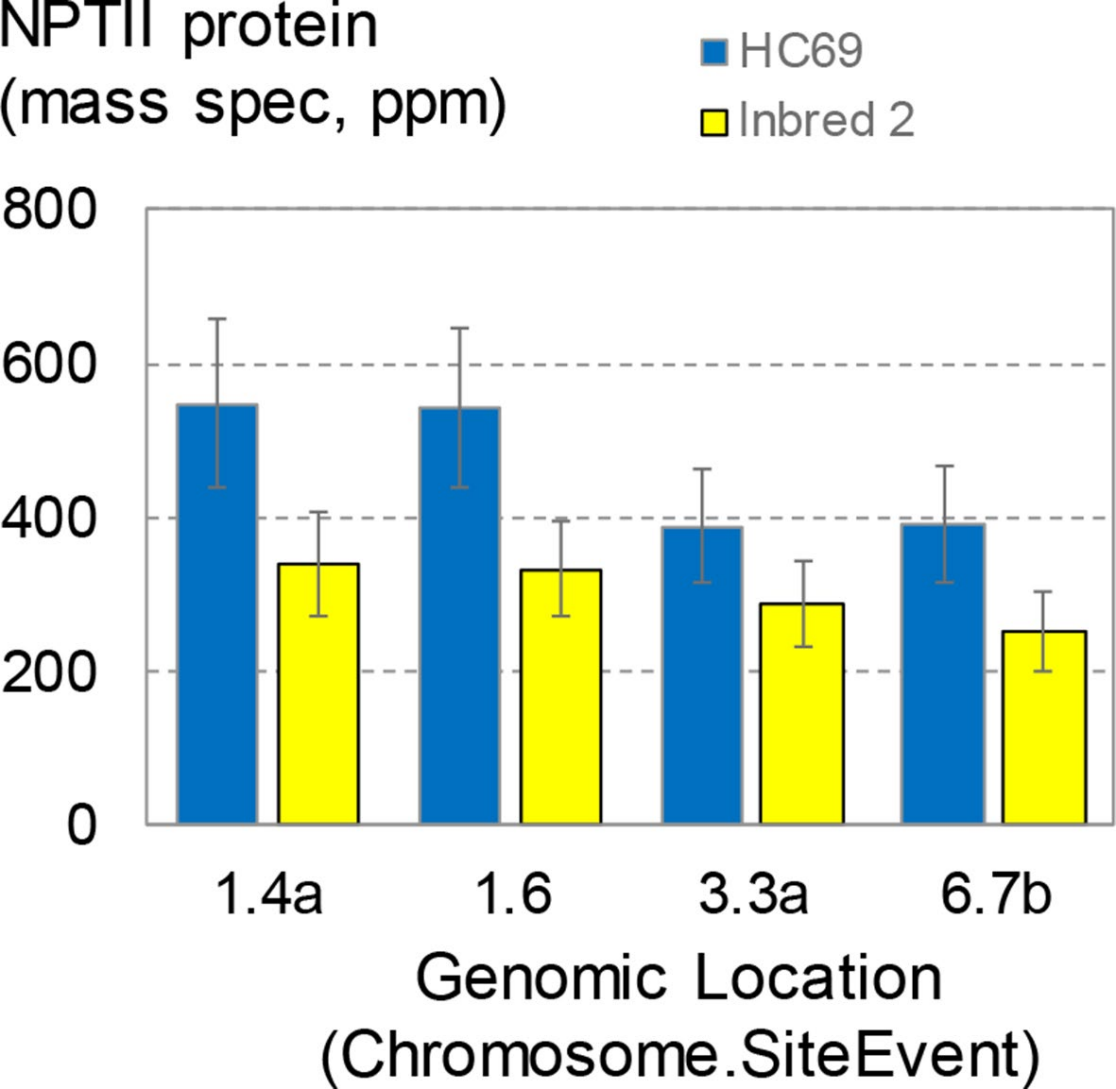
NPTII expression levels in direct transformants and conversions. Four site-specific integration (SSI) landing sites generated by excision-exchange in HC69 inbred maize were introgressed into inbred 2. Completed conversions (>95% recurrent parent) were selfed and seeds homozygous for the transgenic event were produced in the same field and bulked. Bulked seeds were planted in the greenhouse according to the standard complete randomized block design for transgene expression analysis. Young leaves were sampled at about 3 weeks after planting and leaf extracts were analyzed for NPTII concentration. The chart contains data from 114 plants; each bar is the mean of 12-16 plants.

The transgenic maize transformants described so far, including the SSI events, were generated in maize inbred HC69. This inbred was first used as a parent for commercial hybrid seed production in the 1980s. With the goal to establish SSI in a more commercially relevant inbred, designated here as inbred 2, a set of four SSI landing sites in the HC69 genetic background were introgressed by backcrossing to inbred 2. Finished inbred conversions contained >95% inbred 2 (recurrent parent) DNA. Seeds homozygous for the selected SSI landing sites and in the two genetic backgrounds were generated side by side in the field. The four SSI transformants were molecularly identical based on qPCR and SbS analyses. The expression of the nptII transgene at the four insertion sites in the two genetic backgrounds was measured in young leaf tissue following the standard protocol (**Figure 8**). NPTII protein concentrations in young leaves ranged from ∼390 to ∼550 ppm in HC69. The same SSI landing sites introgressed into a different genetic background (inbred 2) accumulated NPTII protein to consistently lower concentrations, with means ranging from ∼250 to ∼340 ppm in young leaves. For three of the four SSI landing sites analyzed, NPTII protein concentrations in HC69 were significantly higher (∼1.6X) compared to the corresponding inbred 2 conversion. By contrast, the highest and lowest expressing SSI transformants in HC69 differed by a factor of ∼2X (**Figure 4A**). Although expression levels among the four sites were not significantly different within each inbred, the same pairs of sites supported higher (1.4, 1.6) and lower (3.3, 6.7) transgene expression levels in both genotypes. This result is consistent with small position effects on transgene expression level.

This dependence of transgene expression level on genetic background has important implications for transgenic event selection for product development. Traditional approaches to transgenic product development have involved generating transgenic events in a single genotype and then screening hundreds to thousands of transformants for one or a few individuals with a preferred, usually high, transgene expression level. The results in **Figure 8** suggest that event sorting based on meeting or exceeding a target expression level in one genetic background may need to account for transgene expression differences following event introgression into different genetic backgrounds. For example, Adamczyk et al. (Adamczyk et al., 2009) found that Cry1Ac protein levels in cotton varied up to about four-fold across five genetic backgrounds following introgression with the same transgenic insertion.

### Most Transgenic Insertion Sites Are Agronomically Neutral

For many, if not all, product development efforts, the fitness of the final organism is critical for commercialization. In maize, we routinely evaluate the impact of transgene expression by measuring, among many other parameters, grain yield, grain moisture, plant and ear height, along with growing degrees units required for pollen shed and silking in hybrid field trials. To evaluate the potential impact of transgene insertions on yield, 32 independent transformants in the HC69 background that contain an SSI landing site (with the structure shown in **Figure 3A**) were used as pollen donors (T4 generation) to make hybrids. As controls for each SSI line, a null segregant at the T2 generation was selected and used to produce T4 plants as pollen donors for hybrid seed production. The 32 SSI sites in five hybrids (160 entries) were evaluated in a multilocation field trial, along with the corresponding controls (additional 160 entries). The SSI landing site encodes the selectable marker nptII, which we had previously observed to be benign with respect to yield (data not shown).

**Figure 9:**
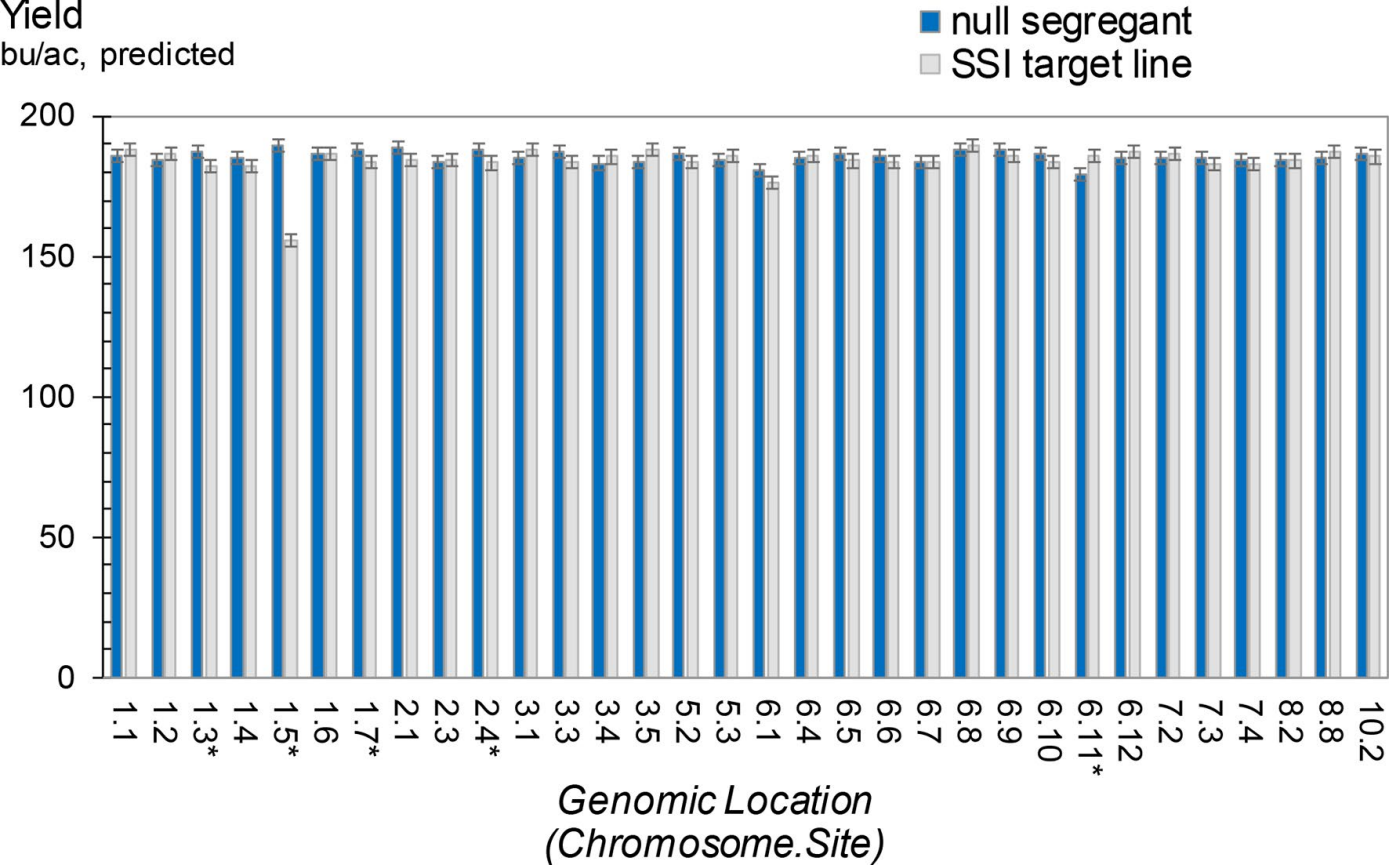
Grain yield for maize hybrids containing a site-specific integration (SSI) landing site. SSI landing sites at 32 unique genomic locations are each compared to a nontransgenic null segregant derived from the same regenerated parent plant. Inbred pollen donors were T3 generation plants either homozygous or null for the indicated SSI site. Pollen donors were top-crossed to five elite line testers to produce F1 hybrid seed for grain yield testing. Yield trials were carried out in a range of optimal and limited-irrigation conditions in the US Corn Belt in 2015. Each bar represents data from five hybrids at up to 16 locations. Asterisk indicates significant differences in yield between a null segregant and corresponding SSI target line.

Average grain yields from the five maize hybrids for each of the 32 SSI target lines and their respective nulls are summarized in **Figure 9**. The trials demonstrated no yield impact attributable to an SSI landing site in 27 of the 32 lines. Five lines showed statistically significant differences between nulls and transgenics, with four nulls yielding higher than their corresponding transgenics and one transgenic yielding higher than its corresponding null. However, only one line showed a large impact on yield. When we further investigated this line through subsequent backcrosses to nontransgenic HC69 combined with phenotypic selections, the yield drag apparently attributable to this SSI landing site was eliminated (data not shown). We therefore conclude that the 32 bu/ac yield drag observed in line 1.5 was not the result of the transgene locus. Although further analysis of the other three lines with minus yield differences is warranted, we predict that these small differences (5-6 bu/ac), although statistically significant (P < 0.05), are likely unrelated to the presence or absence of an SSI landing site *per se* and are likely not reproducible.

## Discussion

A central paradigm in transgenic biology is that chromosomal location, that is, position effect, is an important determinant of transgene expression. Transgenic plant product developers routinely select a single commercial transformant from among hundreds or thousands of sister transformants (Bradford et al., 2005; Burns et al., 2015; Strauss and Sax, 2016). Articles and reviews often refer to “genomic safe harbors” where stable, high-level, and nondeleterious transgene expression can be achieved (Cantos et al., 2014). We have found that genomic location effect on transgenes is a real but minor phenomenon in the important crop plants maize and soybean: transgene expression varies relatively little across most sister transformants at the developmental stages and tissue types tested here. Transgene expression activity was characterized at a total of 51 genomic insertion sites in maize. The 51 sites included 27 independent random T-DNA events plus one to four SSI events located at the sites of each of 24 additional primary T-DNA events. In soybean, 17 SSI transformants at eight random insertion sites were analyzed (five sites were from *Agrobacterium* and three were from particle bombardment). We believe the present report to be the first comprehensive analysis of a large set of sequence-verified single-copy intact insertions using a replicated and randomized experimental design to control for environmental variation in the measurement of transgene expression activity. Because all primary transformants were recovered under selection, the insertion sites characterized here, regardless whether they were recovered using *Agrobacterium* (the majority) or particle bombardment, are limited to those that support the minimum level of expression necessary to survive the particular selection conditions used.

Most insertion sites characterized in this study supported mean transgene expression levels that were within about 30% of all sister transformants, which approaches the experimental error using our methods. This included sets of three to eight random insertions from *Agrobacterium*-mediated transformation as well as sets of one to four SSI transformants at the same landing sites. We are aware of only three previous reports in plants, two in *Arabidopsis* and one in maize, that demonstrated consistent transgene expression among multiple independent transformants. Nagaya et al. (2005) measured transgene expression in T3 generation *Arabidopsis* plants (n = 10) derived from two sets of five sister transformants. The coefficient of variation for transgene expression for the two sets of transformants was 11.9 and 12.6%. De Buck et al. (2004) measured transgene expression in T1 and T2 generation *Arabidopsis* plants (n = 5) and observed variation in the range of two- to four-fold among 19 of 21 events. The other two events expressed the transgene at >10X lower levels and were hypothesized to have been partially silenced. Unger et al. (2001) measured a similar dynamic range in transgene expression level among transformants with multiple constructs in maize. The results presented here demonstrate a similar level of transformant-to-transformant consistency among T1-T4 generation maize and soybean plants with one to two copies of a variety of transgenic insertions. In contrast to numerous published reports (Peach and Velten, 1991; Cocciolone et al., 2000; Day et al., 2000; Cantos et al., 2014), we did not observe expression differences greater than about two- to three-fold among transformants with the same insertion structure. This relatively restricted range of transgene expression variation across genomic sites has been reported previously for SSI sister transformants at three genomic sites in rice (Srivastava et al., 2004; Akbudak and Srivastava, 2017).

Our results and conclusions regarding the importance of insertion site on transgene expression are a departure from what is commonly understood, namely, that insertion site impacts transgene expression, and that only specific portions of the genome are “safe harbors” for transgene insertion. We believe that this apparent discrepancy is largely attributable to different experimental methods used. Some influential studies had significant sources of noise that were often acknowledged as potential confounders, for example, multicopy insertions (Peach and Velten, 1991; Cocciolone et al., 2000), unconfirmed intactness (all studies of multiple sister transformants prior to this one since this is the first known report that includes only transformants with single-copy intact insertions confirmed at the base pair level), studies that used callus or regenerated plants for expression analysis and therefore lack biological replication and statistical power (Peach and Velten, 1991; Cantos et al., 2014). Here we have eliminated or controlled for these confounding factors through replication, randomization, and nucleotide-level molecular characterization.

Our results are also consistent with studies of yeast, where there is little position effect outside of particular regions (e.g., subtelomeric regions and the mating type locus) (Chen et al., 2013; Wu et al., 2017). However, our results are inconsistent with those of studies in insect cells (Markstein et al., 2008) and mammalian cells (Akhtar et al., 2013). More robust techniques for molecular characterization, like SbS, have only recently become practical to perform on large numbers of transformants and precluded precise structural analysis of transgenic insertions until recently. For this reason, differences described in the literature could be attributed in part to technical limitations. However, there are several examples (e.g., Markstein et al., 2008) that use robust experimental designs and convincingly demonstrate reproducible position effect at sites throughout the genome. Therefore, we think a likely explanation is that the importance of position effect varies between different kingdoms and may be largely restricted to Animalia.

Although genomic insertion site has a smaller effect on plant transgene expression than does construct design and genetic background, genomic insertion site can still be important. First, position effect can be used to “fine-tune” expression from a construct whose mean expression level/pattern is not optimal, especially if the transgenic trait will not be introduced into another genetic background, as is the case for vegetatively propagated crops, such as potato, sugarcane, and papaya. Second, if the transgene is to be deployed into multiple genetic backgrounds through backcrossing (trait introgression), genomic regions that are highly conserved may be desirable to increase conversion quality. Furthermore, the data presented here are limited to a subset of available cis-regulatory elements and coding sequences tested in a single developmental stage and in one to two tissue types in two species. Therefore, position effect for certain transformants containing certain transgenes may still impact commercial-level product development that demands transgene efficacy across a wide variety of genetic backgrounds.

The results reported here suggest a certain functional equivalency in transgene expression levels to regions of the maize and soybean genomes accessible to transgene insertion *via Agrobacterium* and particle bombardment. This conclusion raises important considerations for trait engineering in crops. The current transgenic plant development paradigm is to generate a large number (hundreds to thousands) of independent transformants from each DNA construct, followed by extensive screening. Our results suggest that only a small number of high-quality transformants (identified using robust molecular techniques) need to be phenotyped to come to an accurate conclusion with respect to the efficacy and agronomic effects of a transgene. In addition, construct optimization is of paramount importance in transgene expression, including both regulatory sequences and the presence and relative orientation of nearby genes. Last, these results shine a new light on government regulatory paradigms, which are currently centered around event-specific deregulation (Bradford et al., 2005; Strauss and Sax, 2016).

Our results demonstrate that independent transformants are less variable and phenotypically more similar than previously thought both in transgene expression and key agronomically meaningful characteristics. This conclusion suggests that construct-based deregulation may be appropriate. In crops, such as potato, sugarcane, and banana, where trait introgression between varieties is impractical or impossible, a shift to construct-based deregulation by governmental agencies would facilitate product development. None of the transformants we analyzed had any deleterious agronomic performance that could be directly attributed to transgene insertion. These results suggest that successful transgenic product development could be accomplished using a very wide range of insertion sites. Our investigations focused on gene expression using one time point with constitutive, leaf-specific, and root-specific promoters and in controlled environments and broad measures of agronomic performance in the field. Also, transformants were limited to those recovered under selection. There may be genomic locations where inserted transgenes are partially or completely silenced so that the selectable marker gene was not effective. Such sites would not have been detected using our approach but are also not relevant for transgenic trait development. It is possible that we failed to detect minor effects, for example, agronomic impairment under stress conditions or transgene expression variability at a different developmental stage. We also did not target insertions to telomeric or centromeric regions, so we cannot comment on these locations.

Whereas genomic insertion site had a minor effect on transgene expression, we found that many aspects of construct design and genetic background/genotype can have a major impact. As has been reported previously, the choice of promoter has a large impact, as can transcriptional interference from a nearby gene. More surprisingly, we found that transgene expression was highly sensitive to the particular coding region, and the presence of cis-regulatory elements in nonadjacent genes can also have a significant effect on transgene expression. Taken together, these results emphasize the importance and complexity of construct design.

## Materials and Methods

### Constructs and *Agrobacterium*-Mediated Maize Transformation

Constructs used for transformation were built with the following design features. One or more trait gene cassettes (flanked by LoxP sites) were followed by a selectable marker cassette. The selectable marker transgene included a promoter with intron (pZmUbi1) followed by a Flippase Recombination Target (FRT) site, phosphomannose isomerase (PMI) coding sequence, transcriptional terminator (tPINII), and a second FRT site nonidentical to the first. See **Figure S3A** for example T-DNA with LoxP sites flanking the trait cassettes and two FRT sites (indicated by X). Immature embryos of maize inbred HC69 were transformed using *Agrobacterium*, and plants were regenerated according to the method of Cho et al. (2014).

### Determination of Genomic Insertion Sites in Maize

The genomic location of independent T-DNA integration sites was determined by flanking-sequence analysis and using B73 genome sequence as reference. Ligation-mediated-nested PCR (Flanking Seq) utilizes construct-specific PCR primers and Illumina next-generation sequencing technology to characterize transgenic events. The sequence generated from insertional events of interest was used to identify the location of the insertion in the genome, transgene integrity, and transgene copy number. Assay sensitivity and specificity are determined by the nested PCR primer design in which two primers are designed at each border of the construct.

For Flanking Seq analysis, DNA was extracted from lyophilized leaf punches using the EZNA Plate 96 kit (Omega Biotek, Norcross, GA). The isolated DNA was sheared to an average fragment size of 800 base pairs with a Covaris E210 (Covaris Inc., Woburn, MA). The sheared DNA was then end repaired, A-Tailed, and ligated with indexed adapters according to the protocols provided by Kapa Biosystems (Woburn, MA). Following ligation, libraries were enriched for transgene sequences by two rounds of PCR amplifications utilizing nested PCR primers. Following purification with AmpureXP beads (Beckman Genomics, Danvers, MA), enriched libraries were assessed for quality and quantity on the Fragment Analyzer (Advanced Analytical, Ames, IA). The libraries were then pooled in equimolar ratios into a 96-sample pool and then diluted to 2 nM. Pools were sequenced on the Illumina (San Diego, CA) HiSeq 2500 system as 101 bp single-end run per manufacturer protocols.

### Generation of SSI Landing Sites in Maize *via* Cre-Mediated Excision and SSI (Excision Exchange)

SSI landing sites with the structure shown in **Figure 3A** were generated in maize *via* particle bombardment of immature embryos containing a primary transgenic insertion with the structure described in the preceding section (with variable trait cassettes). Converting an integrated T-DNA to an SSI landing site was achieved through two recombinase-mediated reactions—trait gene excision mediated by the Cre-Lox system and cassette exchange mediated by the FLP-FRT system (Gordon-Kamm et al., 2014; Lowe et al., 2016). Immature embryos were cobombarded with the five plasmids shown in **Figure S2**: two plasmids encoded recombinases (Cre, FLP), two encoded morphogenic genes (Babyboom, Wuschel), and one contained the SSI donor DNA flanked by FRT sites matching those in the target event.

Putative excision-exchange transgenic insertions were selected on 100 mg/l G418. qPCR was used to demonstrate removal of the original trait expression cassette(s) and replacement of the selectable marker cassette. qPCR analysis included the genomic flanking sequences 5’ and 3’ of the FRT sites (Flanking Seq analysis described above). qPCR was also used to identify excision-exchange events that had not integrated helper genes Cre, FLP, Babyboom, and Wuschel. The complete and intact structure of the SSI landing sites in individual transformants was confirmed by SbS analysis. Transgenic F1’ generation seeds hemizygous for an SSI landing site and expressing AmCyan were sorted from nontransgenic null segregants (nonfluorescent) prior to planting seed for expression analysis.

### Site-Specific Integration in Maize *via* Particle Bombardment

SSI utilizing SSI landing sites was mediated by particle bombardment following the method in Gordon-Kamm et al. (2014; see Example 3). The donor DNA contained a 5’ FRT site, PMI coding sequence, a double transcription termination sequence (tStPINII::tZm19B1), followed by one or more transgenes of interest, and a 3’ FRT site. For an example, see **Figure 7A** where the SSI donor sequence corresponds to the DNA between the two FRT sites identified by X. A plasmid containing the SSI donor DNA was cobombarded with the three plasmids encoding FLP, Wuschel, and Babyboom (**Figure S2**). Selection for PMI activity was as described in Cho et al. (2014) using 12.5 g/L mannose, 5 g/L maltose. Regenerated plants were screened by qPCR for the intended SSI outcome at the promoter trap locus (e.g., **Figure 6B**, **Figure 7A**) and for the absence of FLP or either of the morphogenic genes. Regenerated T0 SSI plants were selfed and/or pollen was carried to silk on wild-type plants to generate T1 and F1’ seeds, respectively.

### Soybean Transformation *via* Particle Bombardment or *Agrobacterium*

Transgenic soybean (*Glycine max*, cultivar 93B86) plants were produced using embryogenic cultures as explants (Finer and McMullen, 1991; Stewart et al., 1996; Li et al., 2007). Briefly, soybean embryogenic suspension cultures were generated as described by Samoylov et al. (1998). The cultures were maintained in 250-ml flasks containing 50 ml of liquid media on rotary shakers at 26°C under cool white fluorescent lights with a 16/8-h day/night photoperiod. To create SSI landing sites, fresh embryogenic cultures were bombarded with 0.6µ gold particles coated with the *Ava*I fragment shown in **Figure 3B**. A biolistic instrument PDS1000/HE (Bio-Rad, Hercules, CA) (Klein et al., 1987; Li et al., 2007) was used for these experiments. Similarly, *Agrobacterium tumefaciens* AGL-1 carrying a binary plasmid with the FRT landing sites (**Figure 3B**) was used to transform embryogenic cultures (Cho et al., 2011). Transgenic events were selected using 30 µg ml ^-1^ hygromycin. Later, T0 plants were regenerated and transferred to growth chambers. Single-copy transgenic insertions were identified by qPCR (Li et al., 2007) and confirmed by SbS (Zastrow-Hayes et al., 2015). In this study, three SSI landing sites were developed using the particle bombardment method (7.1, 7.2, and 9.2), and five SSI sites were developed using the *Agrobacterium*-mediated transformation process (5.1, 9.1, 10.4, 12.1, and 19.5). The chromosomal locations of each of these SSI sites were determined by comparing the flanking sequence obtained by SbS to the genomic sequence of 93B86. The chromosomal locations were later reconfirmed by comparing the physical and genomic maps using marker-assisted backcrossing (unpublished data).

T0 plants were grown to maturity under the same conditions as the wild-type plants but in separate growth chambers. T1 seeds were harvested and later planted to identify homozygous transgenic plants using qPCR and confirmed by SbS analysis.

### Site-Specific Integration in Soybean *Via* Particle Bombardment

Homozygous T1 soybean plants containing one of the eight SSI landing sites described above were used as starting material for SSI (Li et al., 2009; Li et al., 2010). Similar to the above particle bombardment process, embryogenic cultures were cobombarded with donor circular vector and FLP expression plasmid (pGmEF1A:FLP:tStPINII) at a DNA concentration ratio of 9:3. The structure of the the resulting SSI event following exchange between the FRT sites is shown in **Figure 3B**. Selection of SSI events was done on 90 ng ml^-1^ chlorsulfuron (DuPont; Li et al., 2009; Li et al., 2010). SSI events were confirmed by qPCR and SbS. Regenerated (T0) SSI events at eight genomic locations were grown to maturity in growth chambers. T1 seeds were harvested and later planted to identify homozygous transgenic plants using qPCR and confirmed by SBS. T2 seeds were separately collected from individual homozygous plants for each of the eight SSI landing sites.

### Field Production of Maize Seed From Sequential Generations

Seed homozygous for a transgenic insertion of interest was produced in field plots using the ear-row selection method. Leaf tissue samples were collected from T2 generation plants and transgene zygosity was determined by qPCR. Plants homozygous for the transgene of interest were selfed by hand pollination. The resulting T3 generation seed was harvested as individual ears. To produce T4 seeds, the three best ears were planted for seed increase. Two rows were planted for each of the three ears with a minimum of 15 seeds per row and were tested for the presence of the transgene of interest by qPCR analysis. During the T3 generation, five ears bearing T4 generation seeds were harvested and shelled individually for future seed increases, and the remaining ears were bulk shelled. T4 and T5 generations followed the same procedure, planting a minimum of three ears, assigning a breeder score ranking the ear rows and harvesting five ears to be individually shelled and bulk harvesting the remaining plants for expression and other testing needs.

### Experimental Design for Transgene Expression Analysis of Maize Leaf and Root

A randomized block design was used to grow maize plants in the greenhouse for collection of leaf and root samples for analysis of transgene transcript levels and recombinant protein concentration. Power analysis after a pilot study was performed to determine sufficient sample sizes at 80% power with 0.05 type I error rate. Subsequent experiments all have a sample size of at least 16 to ensure 80% power. Unless indicated otherwise, F1 seeds hemizygous for a transgenic event were selected visually based on expression of a fluorescent protein, and null segregant (nonfluorescent) seeds were discarded. Transgenic seeds from individual ears or bulked seed from field plots were planted in four replicates of four to eight kernels. Each replicate was planted in a single row (four seeds) or two adjacent rows (eight seeds) in rectangular flats (8 rows x 4 columns). Each flat therefore contained four or eight replicates depending on the number of seed per replicate. Deviations from this basic design (e.g., 8-12 reps of four seeds) are described in the figure legends. Flats were placed side by side on tables in the greenhouse.

### Growth and Sampling of Maize Plants

Planting and sampling of maize plants were performed by teams of researchers to minimize elapsed time and any variation due to circadian effects on physiological status of leaves and roots. Randomization of replicates across the experimental footprint helped control for any residual variation attributable to physiological status changes during the tissue sampling window. Individual experiments were planted on the same day and sampled on the same day. Maize seeds were sown at a 2.5-cm depth into 32-cell flats (90.7-ml individual cell volume) containing a soil-less substrate composed of (by vol.) 77% Canadian sphagnum peat, 16% perlite, and 7% vermiculite and adjusted with lime to a pH of 6.1 and irrigated with municipal water supplemented with 125 mg·L–1 N (Peters Excel ^©^ Cal-Mag ^©^ 15N–2.2P–12.5K Everris NA, Marysville, OH). Plants that were sampled for root material were grown in Turface (Profile Products LLC Mfg. Number: BFMVP5004). Seeds were germinated and grown in a greenhouse with environmental set points shown in **Table S2**. The greenhouse set-points were programmed and maintained continuously (Argus Control Systems, Ltd., Surrey, British Columbia).

At V3 plant stage (third leaf is the uppermost leaf with collar visible), plants were sampled for either root material or leaf material. Sampling was typically completed in 2-4 h between 8:00 AM and noon. Leaf material was sampled for quantitative protein analysis by collecting six leaf punches (6.35-mm diameter) from the uppermost leaf with a fully developed and visible leaf collar, avoiding the midrib and edges of the leaf. Samples were collected and stored on dry ice using 1.2-ml microtiter tube (Thermo Fisher #2681376) inserted into prechilled Deep-Well sampling plates (Thermo Fisher #P9635FIS). At no time were the samples allowed to freeze/thaw prior to analysis.

Root material for quantitative protein analysis was collected on V3 stage plants by removing the entire plant from Turface material, dipping in clean DI water to remove any substrate material clinging to the roots, and sampling with scissors. A root sample was obtained by cutting ∼1.5 cm from both the radicle and lateral seminal roots consisting of three to four pieces and placing them into a 1.2-ml microtiter tube inserted into the Deep-Well sampling plates prechilled on dry ice.

### Growth and Sampling of Soybean Plants

T2 generation soybean seeds from 17 SSI transformants (representing eight SSI landing sites) and seed for soybean variety 93B86 (wild-type control) were planted in flats in the greenhouse. T2 homozygous transgenic SSI plants and their null segregants were identified using qPCR from leaf tissue. For each of the 17 SSI events, 10 healthy homozygous plants were selected as replicates along with null-segregant sibs and 93B86 wild-type plants as controls. Plants were transplanted to 8-inch pots.

All plants were grown in a single greenhouse room on three benches. A split-plot experimental design was used. Main plot: events were randomized across replicates. Subplot: segregation (positive/negative nulls) nested within events. When the plants reached V5 stage (fifth trifoliate fully expanded), leaf tissue punches from the V1 leaf of all plants in this experiment were collected and immediately frozen prior to processing for mass spec analysis. Concentrations of GAT protein in leaf extracts were measured by mass spec analysis (see Protein Analysis below).

### Molecular Characterization of Transgenic Maize Plants

Transgenic insertions in regenerated plants and their progeny were detected by qPCR and SbS using leaf tissue samples according to the method of Zastrow-Hayes et al. (2015).

### Protein Analysis (Mass Spectroscopy)

Extraction and Digestion: Proteins assayed by mass spectroscopy were extracted from 10 mg of lyophilized leaf material similar to previously published protocol for ARGOS8 (Schacherer et al., 2017) with some minor modifications. A dry grind was added prior to the addition of extraction buffer using 2 x 30-s cycles for both the dry and wet grinds for all proteins. Extraction buffers varied per protein (ARGOS8 100 mM ammonium bicarbonate with 0.1% Triton X-100 and 0.5% CHAPS; NPTII 8 MUrea with 5 mM DTT and 0.05% Tween-20; PBST for GAT, PAT, PMI, and ALDH7). All samples were normalized to a fixed total protein concentration based upon Bradford assay results (Bradford Protein Assay Kit, Bio-Rad). Purified protein standard curve prepared in null leaf extract and controls (null and a known positive) were included on each assay plate. All samples (50 µl) were mixed with 100 µl buffer (100 mM ammonium bicarbonate with 0.05% Tween-20) followed by incubation with DTT (6 µl 250 mM) at 50°C (exception 95°C for ARGOS8) for 30 min, followed by the addition of IAA (6 µl 300 mM) with incubation for 30 min at room temperature in dark followed by the addition of a specific concentration of trypsin (1 µg for ALDH7, PAT, PMI, NPTII, GAT; 2 µg for ARGOS8) with incubation at 37°C for ∼18 h. All samples received 10% formic acid (10 µl) and the addition of a heavy isotope labeled internal standard (same sequence as the tryptic peptide of interest) prior to LC-MS/MS analysis. All samples were centrifuged briefly following each reagent addition (30 s at 1,200 rpm).

LC-MS/MS: Conditions were similar to those used in a previously published protocol for ARGOS8 (Schacherer et al., 2017) with some modifications for the other proteins. Mobile phases consisted of 0.1% formic acid (MPA) and 0.1% formic acid in acetonitrile (MPB). All MPB started for 0.1 min, followed by a 1.5-min linear gradient to the final higher MPB gradient target. For ALDH7, PAT, and PMI, the LC run started at 15% MPB, and the gradient ended at 25% MPB. For NPTII, the LC run started at 10% MPB, and the gradient ended at 40% MPB. For GAT, the LC run started at 2% MPB, and the gradient ended at 31% MPB.

The mass spectrometer was run in MRM mode at unit-mass resolution for both Q1 and Q3. For ZmARGOS8, the following source conditions were used: dwell time, 50 ms; curtain gas, 30 psi; ion spray voltage, 1,900 V; ion source temperature, 550°C; ion-source gas 1, 70 psi; ion-source gas 2, 80 psi; collision gas, medium. For all other proteins, the following conditions were used: dwell time, 50 ms; curtain gas, 30 psi; ion spray voltage, 2,000 V; ion source temperature, 600°C; ion-source gas 1, 50 psi; ion-source gas 2, 50 psi; collision gas, medium. **Table S3** provides information on the tryptic peptide sequence and the MRM transitions for each. Results were reported as parts per million based on total protein.

### Protein Analysis (ELISA)

Leaf punches (four) were extracted in PBST (500 µl) with two metal beads at 1,650 rpm for 60 s followed by centrifugation (4°C, 3,889 x g) for 10 min. A Bradford total protein assay was performed (Bradford Protein Assay Kit, Bio-Rad). Polyclonal based sandwich ELISA assays were developed following the methods previously published (Schmidt and Alarcon, 2010). IP2-127 specific polyclonal antibody assay standard curve range was 0.5 ng/ml to 10 ng/ml. IPD032-specific polyclonal antibody assay standard curve range was 1 to 20 ng/ml. Samples were assayed in duplicate with comparison of interpolations across varying sample dilutions. Controls (negative and known low and high positive) were included on each assay plate. Results were reported as parts per million based on total protein.

### Statistical Analysis of Expression

Expression studies were analyzed using linear mixed models. The model includes variables that contribute to technical and biological variations. Technical variations include spatial variability due to the location of each plant in the greenhouse, batch effects that correlate to outcome of interest such as personnel, tissue sampling, laboratory conditions, and total protein concentration. Biological variations include genetic background, family, genetic location, and other variables, such as generation and transformation type that are relevant only for a subset of these studies. Pairwise t-tests were applied after model fit to determine significant differences among the variables of interest. All analyses were done using R. For all events analyzed for transgene expression, only events with samples obtained from at least 10 individual plants were included in the analysis.

### Generation of F1 Hybrid Seed for Field Testing

Regenerated (T0 generation) plants containing excision-exchange transformants with the SSI landing site structure shown in **Figure 3A** were selfed for two successive generations to develop T2 generation seed segregating the transgenic SSI landing site. In the T2 generation for each transformant, a plant homozygous for the SSI landing site and a null segregant plant were identified by zygosity qPCR targeting the nptII transgene. Selected T2 plants were selfed to produce T3 generation seeds. In the T3 generation, individual ears were selected for uniformity and on-type phenotype. Selected ears were top-crossed to five elite line testers for yield testing. F1 hybrid seed were therefore either null or hemizygous for an SSI target site.

### Hybrid Yield Testing

Hybrid yield testing was conducted in 13 optimal and 3 limited-irrigation locations in the United States. The experimental design was two-row plot nested by tester and a set of transformants and corresponding null segregants as controls. The transformants contained an SSI landing site with an nptII_AmCyan molecular stack (**Figure 3A**). The null-segregant comparators were derived as described above. One replication augmented with diagonal checks (wild-type reference) per location. The transformants were each compared to null-segregant controls, and statistical differences were determined at *P* < 0.05.

### Statistical Models

#### Normal Location

EU_Multiloc_NEST_Pfbg_Ctl_Site_Pftsbg_Concept_Evt_Fam_Evbg_Diag

A linear mixed model was applied to model yield for cross locations. Data for yield (*Y* *_igbmnfcs_*) of location (*L*)*i*, platform (*G*)*_g_*, background (*B*)*_b_*, insertion site (*S*)*_m_*, event (*E*)*_n_*, family (*F*)*_f_*, chromosomal region (*C*)*_c_*, and plot *s* were modeled as a function of an overall mean μ, factors for location, platform, background, location by platform, location by background, insertion site, insertion site by event, location by insertion site, location by insertion site by event, background by insertion site by event, location by background by site by event, family, family by chromosomal region, location by family, location by family by chromosomal region, platform by family, platform by family by chromosomal region, background by family, background by family by chromosomal region, location by platform by family, location by platform by family by chromosomal region, location by background by family, location by background by family by chromosomal region, event by family, location by event by family, background by event by family, location by background by event by family and a residual within each location (ε/*L*)*_igbmnfcs_*. The model can be specified as:

$$\begin{array}{l} \begin{array}{l} Y_{igbmnfcs}=µ+\underline{L_{i}}+\underline{G_{g}}+\underline{B_{b}}+\underline{{\left(L\times G\right)}_{ig}}+\underline{{\left(L\times B\right)}_{ib}}\\ \;+S_{m}+\underline{{\left(S\times E\right)}_{mn}}+\underline{{\left(L\times S\right)}_{im}} \end{array}\\ \begin{array}{l} \;+\underline{{\left(L\times S\times E\right)}_{imn}}+\underline{{\left(B\times S\times E\right)}_{bmn}}\\ \;+\underline{{\left(L\times B\times S\times E\right)}_{ibmn}}+F_{f}+(F\times C{)}_{fc} \end{array}\\ \begin{array}{l} \;+\underline{{\left(L\times F\right)}_{if}}+\underline{{\left(L\times F\times C\right)}_{ifc}}+\underline{{\left(G\times F\right)}_{gf}}\\ \;+\underline{{\left(G\times F\times C\right)}_{gfc}}+\underline{{\left(L\times G\times F\right)}_{igf}} \end{array}\\ \begin{array}{l} \;+\underline{{\left(L\times G\times F\times C\right)}_{igfc}}+\underline{{\left(B\times F\right)}_{bf}}\\ \;+\underline{{\left(B\times F\times C\right)}_{bfc}}+\underline{{\left(L\times B\times F\right)}_{ibf}} \end{array}\\ \begin{array}{l} \;+\underline{{\left(L\times B\times F\times C\right)}_{ibfc}}+\underline{{\left(E\times F\right)}_{nf}}\\ \;+\underline{{\left(L\times E\times F\right)}_{inf}}+\underline{{\left(B\times E\times F\right)}_{bnf}} \end{array}\\ \;+\underline{{\left(L\times B\times E\times F\right)}_{ibnf}}+\underline{{\left(\varepsilon /L\right)}_{igbmnfcs}} \end{array}$$

Where site, family, and family by chromosomal region were treated as fixed effect, and all the other effects except the residual were treated as independent normally distributed random variables with means of zero. For the residual, instead of assuming independence among plots, two-dimensional separable first-order autoregressive correlation (AR1 X AR1) structure was applied to capture plot-to-plot correlations in both row and column directions of the field besides the plot-to-plot variation. *T*-tests were conducted to compare treatment effects. A difference was considered statistically significant if the *P*-value of the difference was less than 0.05. All data analysis and comparisons were made in ASReml 3.0 (VSN International, Hemel Hempstead, UK, 2009).

#### Stress Location

EU_Multiloc_NEST_pfbg_ctl_site_pftsbg_concept_evt_fam_evbg

#### Normal and Stress Location

EU_Multiloc_NEST_pfbg_ctl_site_pftsbg_concept_evt_fam_evbg_diag_rep

(model dealt with diagonal check, did not reflect it in the description below)

A linear mixed model was applied to model yield for cross locations. Data for yield (*Y* *_igjbmnfcs_*) of location (*L*)*_i_*, platform (*G*)*_g_*, replication (*R*)*_j_*, background (*B*)*_b_*, site (*S*)*_m_*, event (*E*)*_n_*, family (*F*)*_f_*, chromosomal region (*C*)*_c_*, and plot *s* were modeled as a function of an overall mean μ, factors for location, platform, location by platform, location by platform by replication, background, location by background, location by replication by background, site, site by event, location by site, location by site by event, location by replication by site by event, background by site by event, location by background by site by event, location by replication by background by site by event, family, family by chromosomal region, location by family, location by family by chromosomal region, platform by family, platform by family by chromosomal region, background by family, background by family by chromosomal region, location by platform by family, location by platform by family by chromosomal region, location by background by family, location by background by family by chromosomal region, event by family, location by event by family, background by event by family, location by background by event by family, and a residual within each location (ε/*L*)*_igjbmnfcs_*. The model can be specified as:

$$\begin{array}{l} \begin{array}{l} Y_{igjbmnfcs}=µ+\underline{L_{i}}+\underline{G_{g}}+\underline{{\left(L\times G\right)}_{ig}}+\underline{{\left(L\times G\times R\right)}_{igj}}\\ \;+\underline{B_{b}}+\underline{{\left(L\times B\right)}_{ib}}+\underline{{\left(L\times R\times B\right)}_{ijb}} \end{array}\\ \begin{array}{l} \;+S_{m}+\underline{{\left(S\times E\right)}_{mn}}+\underline{{\left(L\times S\right)}_{im}}\\ \;+\underline{{\left(L\times S\times E\right)}_{imn}}+\underline{{\left(L\times R\times S\times E\right)}_{ijmn}} \end{array}\\ \begin{array}{l} \;+\underline{{\left(B\times S\times E\right)}_{bmn}}+\underline{{\left(L\times B\times S\times E\right)}_{ibmn}}\\ \;+\underline{{\left(L\times R\times B\times S\times E\right)}_{ijbmn}}+F_{f} \end{array}\\ \begin{array}{l} \;+(F\times C{)}_{fc}+\underline{{\left(L\times F\right)}_{if}}+\underline{{\left(L\times F\times C\right)}_{ifc}}\\ \;+\underline{{\left(G\times F\right)}_{gf}}+\underline{{\left(G\times F\times C\right)}_{gfc}} \end{array}\\ \begin{array}{l} \;+\underline{{\left(L\times G\times F\right)}_{igf}}+\underline{{\left(L\times G\times F\times C\right)}_{igfc}}\\ \;+\underline{{\left(B\times F\right)}_{bf}}+\underline{{\left(B\times F\times C\right)}_{bfc}} \end{array}\\ \begin{array}{l} \;+\underline{{\left(L\times B\times F\right)}_{ibf}}+\underline{{\left(L\times B\times F\times C\right)}_{ibfc}}\\ \;+\underline{{\left(E\times F\right)}_{nf}}+\underline{{\left(L\times E\times F\right)}_{inf}} \end{array}\\ \begin{array}{l} \;+\underline{{\left(B\times E\times F\right)}_{bnf}}+\underline{{\left(L\times B\times E\times F\right)}_{ibnf}}\\ \;+\underline{{\left(\varepsilon /L\right)}_{igjbmnfcs}} \end{array} \end{array}$$

Where site, family, and family by chromosomal region were treated as fixed effect, and all the other effects except the residual were treated as independent normally distributed random variables with means of zero. For the residual, instead of assuming independence among plots, two-dimensional separable first-order autoregressive correlation (AR1 X AR1) structure was applied to capture plot-to-plot correlations in both row and column directions of the field besides the plot-to-plot variation. *T*-tests were conducted to compare treatment effects. A difference was considered statistically significant if the *P*-value of the difference was less than 0.05. All data analysis and comparisons were made in ASReml 3.0 (VSN International, Hemel Hempstead, UK, 2009).

### Materials Availability

Novel biological materials described in this publication may be available to the academic community and other not-for-profit institutions solely for noncommercial research purposes upon acceptance and signing of a material transfer agreement between the author’s institution and the requestor. In some cases, such materials may originally contain genetic elements described in the manuscript that were obtained from a third party, and the authors may not be able to provide materials including third-party genetic elements to the requestor because of certain third-party contractual restrictions placed on the author’s institution. In such cases, the requester will be required to obtain such materials directly from the third party. The authors and authors’ institution do not make any express or implied permission(s) to the requester to make, use, sell, offer for sale, or import third-party proprietary materials. Obtaining any such permission(s) will be the sole responsibility of the requestor. To protect Corteva Agriscience's proprietary germplasm, such germplasm will not be made available, except at the discretion of Corteva Agriscience and then only in accordance with all applicable governmental regulations.

## Accession Numbers

### 5’ Regulatory Sequences

pZmUbi, S94464 [5’ regulatory sequence only; (Christensen et al., 1992)]

### Coding Sequences

pZmARGOS8, JN252302 [coding sequence only; (Shi et al., 2015)]

## Data Availability Statement

The datasets generated for this study will not be made publicly available. Some data, for example, describing transgene insertion sites at the DNA sequence level, may be proprietary.

## Author Contributions

DP, BL, CS, EW, HG designed, generated, and analyzed maize transformants. JB, RB, SC, ZL, AK designed, generated, and analyzed soybean transformants. CS, JF, SB, SDB, AC, NC, DS, LL, XS designed maize expression experiments. XS developed statistical models and performed final statistical analyses for expression studies. KH coordinated protein concentration analyses. BL, CS, JS, JF, SDB performed and/or coordinated molecular analyses for maize. MM, JM and XS designed and analyzed expression experiment for maize conversions. JM designed, executed and analyzed the maize yield experiments. LL developed statistical model for yield trials. MM and JM did the ear-row selections, seed handling, maintenance and storage of all seeds from T1 to TN generations. SDB and NC wrote the first draft of the manuscript. XS, JM, MM, KH, JF, JB, DP, BL, CS wrote sections of the manuscript and/or helped prepare drafts of some figures. SDB, NC, XS, JS, AC revised the manuscript. All authors read and approved the submitted version. 

## Conflict of Interest

All authors were employees of Corteva Agriscience at the time of their contributions to any experimental work. During the writing and revising of the manuscript, AMC was employed by Genus, ZL was employed by Kenfeng Seed, and SC was employed by Benson Hill Biosystems.

The remaining authors declare that the research was conducted in the absence of any commercial or financial relationships that could be construed as a potential conflict of interest.

## Abbreviations

FLP, flippase recombinase; FRT, FLP recombinase target; ORF, open reading frame; PCR, polymerase chain reaction; SbS, Southern by Sequencing; SSI, site-specific integration.
