# Supplementary material for: Uniform Expression and Relatively Small Position Effects Characterize Sister Transformants in Maize and Soybean
Source: Front Plant Sci. 2019 Oct 24;10:1209. doi: 10.3389/fpls.2019.01209 (PMC6821721; doi:10.3389/fpls.2019.01209)
Supplement: Supplementary file 1 [file DataSheet_1.pdf]

Figure S1. Detection of a partial transgene deletion in a T-DNA 3 event identified as single copy and intact by qPCR. RB, right T-DNA border; LB, left T-DNA border; 6x@, stop codons in all 6 reading frames; p, promoter element, t, terminator element; ∇, LoxP site; X̲, FRT site. Arrows indicate the direction of transcription. Top: PAT protein concentrations in leaf extracts. Bottom: SbS analysis of ~15 kb T-DNA region in an example complete event and event DR containing a partial deletion at the 5' end.

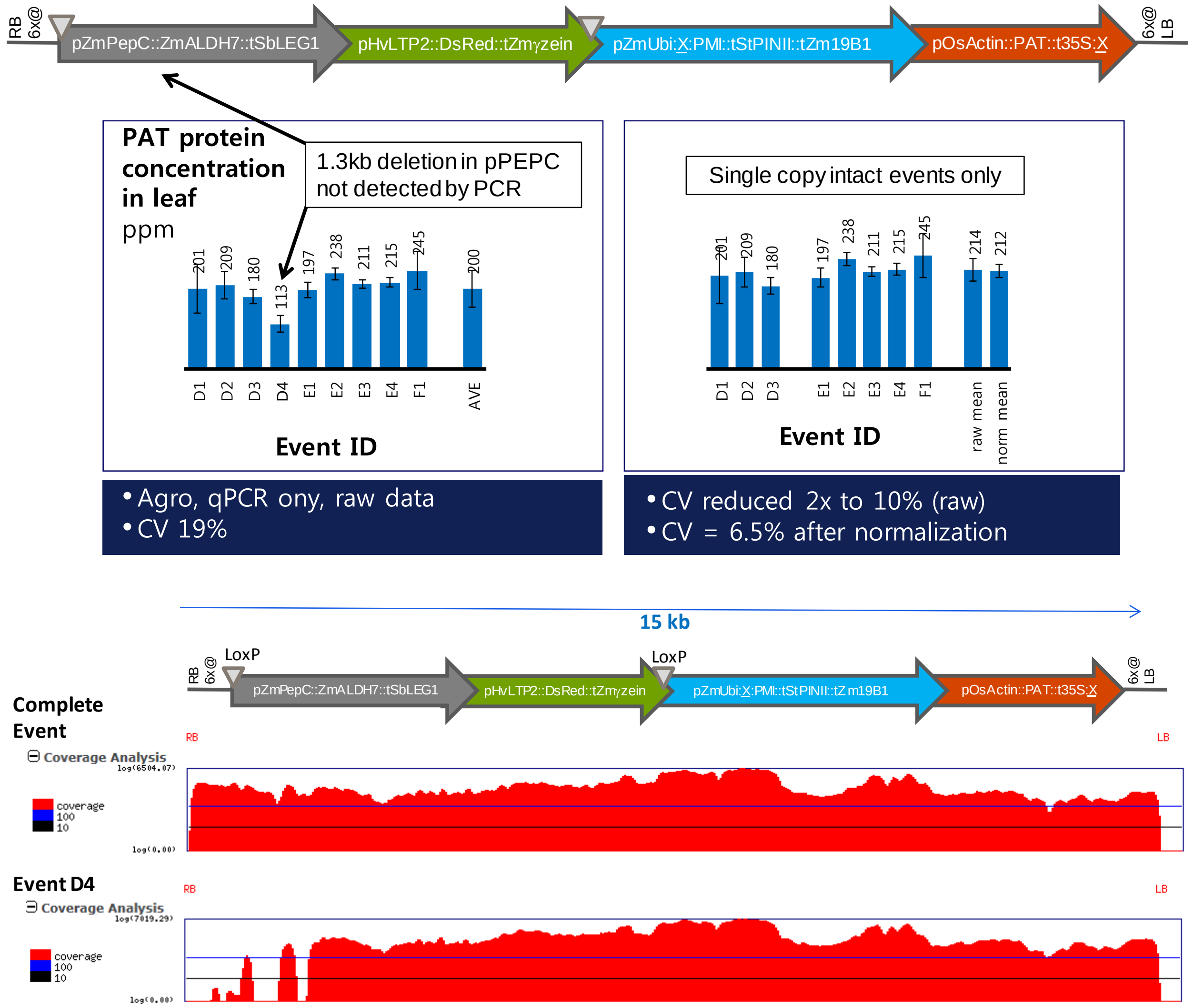

Figure S2. Plasmids used for excision-exchange to generate SSI landing sites in maize.

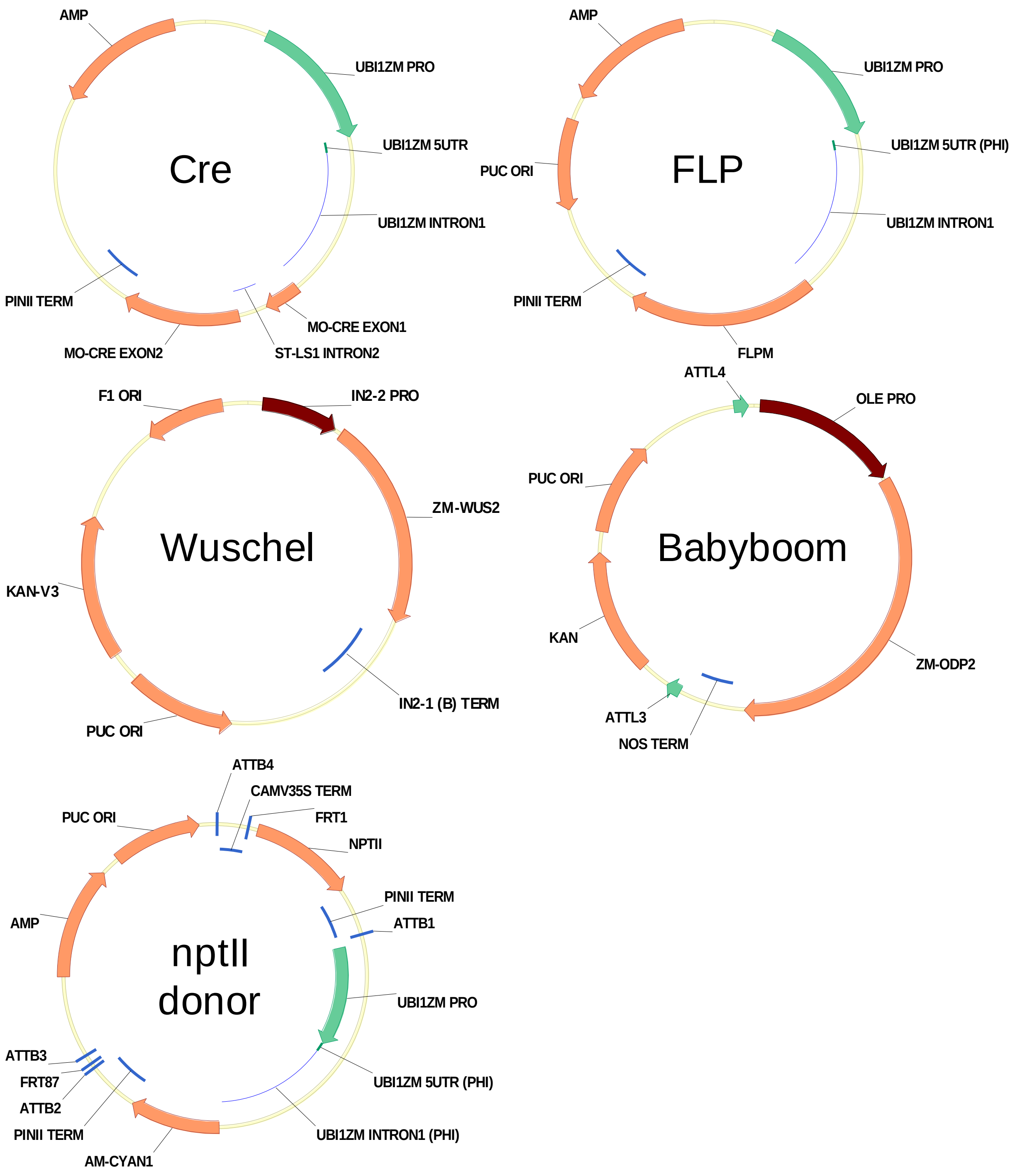

Figure S3. Structures of SSI landing site intermediates and SSI landing sites in maize and soybean. LoxP, Cre recombinase target site; X, FRT site (FLP Recombinase Target site); RB, right border; LB, left border; 6x@, stop codons in all six reading frames. **A**. Structure of precursor SSI landing site in maize. As described in Methods, transgenic events with the structure shown here were modified by Cre-mediated excision of the LoxP-LoxP fragment containing one or more trait gene(s) followed by FLP-FRT-mediated cassette exchange to replace the fragment X-X and generate the SSI landing site shown in Figure 3A. **B**. SSI landing site in soybean generated by Agro-mediated transformation. **C**. SSI landing site in soybean generated by particle bombardment transformation.

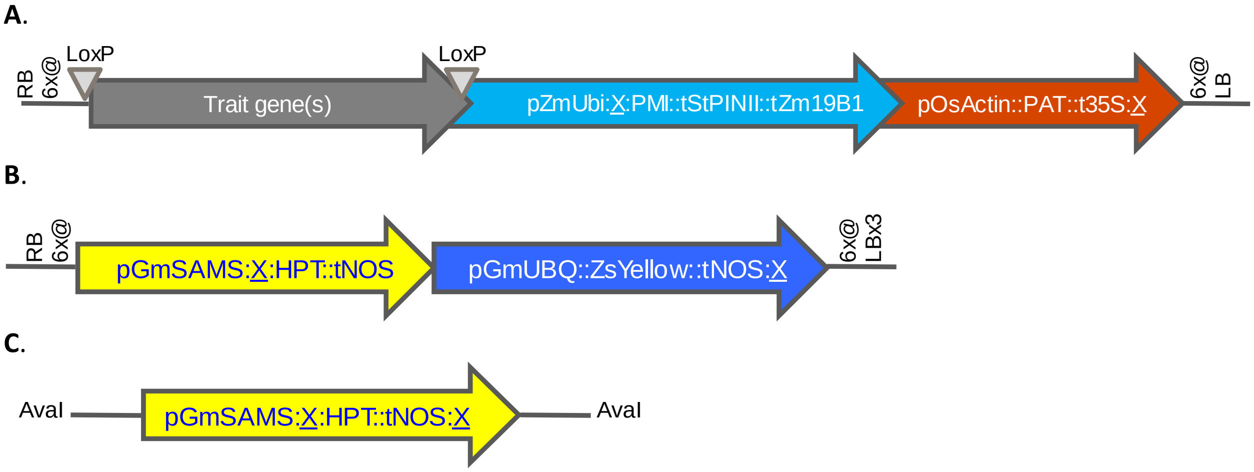

Table S1. List of DNA regulatory elements and coding sequences present in transgene cassettes.

Function	Abbreviation	Name of source gene or gene product	Source species
Coding Sequences	ALDH7	antiquitin-like aldehyde dehydrogenase	*Zea mays*
	AM-CYAN	blue fluorescent protein (gene from Clontech)	*Anemonia majano*
	ARGOS8	auxin regulated gene involved in organ size	*Zea mays*
	CTP_IP2-127	chimeric chloroplast transit peptide from maize and rice genes fused with an engineered variant of the cry2Ab gene	*Bacillus thuringiensis*
	DsRed	a variant of Discosoma sp. red fluorescent protein (DsRed), from Clontech 'BD Living Colors'	*Discosoma* species
	GAT	glyphosate acetyltransferase	*Bacillus licheniformis*
	HPT	hygromycin phoshotransferase	*Escherichia coli*
	IPD032	Insecticidal Protein Discovery gene number 32	*Alcaligenes faecalis*
	NPTII	neomycin phosphtransferase II	*Escherichia coli*
	PAT	maize-optimized phosphinothricin acetyltransferase	*Spectomyces hygroscopicus*
	PMI	phosphomannose isomerase	*Escherichia coli*
	ZsYellow	yellow fluorescent protein from Clontech	*Streptomyces hygroscopicus*
Hairpin transcripts	VRS1-IR	Transcript produces RNA hairpin comprised of sense and anti-sense versions of a 532-bp 5'-truncated fragment from the maize vulgare six-rowed spike protein separated by a 249 bp sequence containing the 189 bp intron 2 from the potato LS1 gene	*Zea mays, Solanum tuberosum*
5' regulatory	pAtUbi	ubiquitin	*Arabidopsis thaliana*
	pAtUBQ10	ubiquitin 10	*Arabidopsis thaliana*
	pBSV	banana streak virus promoter plus intron from maize ortholog of a rice hypothetical protein	Banana streak virus, *Zea mays*
	pGmSAMS	S-adenosyl-L-methionine synthetase 5' regulatory region including 5' untranslated region and first intron	*Glycine max*
	pGmUBQ	hexameric polyubiquitin 5' regulatory region including 5' untranslated region and first intron (PSO333229)	*Glycine max*
	pHvLTP2	lipid transfer protein 2	*Hordeum vulgare*
	pOsActin	actin 1 promoter plus first intron	*Oryza sativa*
	pSbUbi	ubiquitin	*Sorghum bicolor*
	pZmPEPC	phospoenolpyruvate carboxylase	*Zea mays*
	pZmRCC3	root cortical cell delineating protein	*Sorghum bicolor*
	pZmUbi	ubiquitin 1 promoter plus 5' untranslated region and first intron	*Zea mays*
3' regulatory	t35S	35S	Cauliflower mosaic virus
	tAtUBQ3	ubiquitin-3	*Arabidopsis thaliana*
	tAtRPG	ribosomal protein gene	*Arabidopsis thaliana*
	tOsUbi	ubiquitin	*Oryza sativa*
	tPvV2	reserve globulin	*Phaseolus vulgaris*
	tSbGKAF	gamma-kafirin storage protein	*Sorghum bicolor*
	tSbLEG1	legumin 1	*Sorghum bicolor*
	tStPINII	proteinase inhibitor II	*Solanum tubersum*
	tZm19B1	19-kD B1 zein	*Zea mays*
	tZmγzein	27 KD gamma zein	*Zea mays*

Table S2. Maize growth conditions. Day after sowing, day temperature, night temperature, light integral, day length and vapor pressure were recorded from sowing until 21 days post sowing.

Days after Sowing	Day Temp (° C)	Night temp (° C)	Daily Light Integral ($\mu mol \cdot m^{-2} \cdot d^{-1}$)	Day Length (hrs)	Vapor Pressure Deficit (mbar)
0-7	28.3	25.5	15	16	7
8-14	25.5	22.7	20	16	8
15-21	26.1	21.1	25	16	10

Table S3. Protein specific tryptic peptide sequence information, location of heavy isotope labeled amino acid*, and MRM transitions.

Protein	Peptide Sequence	Q1	Q3
ARGOS8	ALALGQVSVMR	572.9	776.3
ARGOS8 IS	ALALGQVSV*MR	575.9	782.3
PAT	SVVAVIGLPNDPSVR	762	784.3
PAT IS	SVVAVIGL*PNDPSVR	765.5	784.3
ALDH7	IPLVSFTGSTR	589.3	755.3
ALDH7 IS	IPLVSFTG*STR	590.8	758.3
NPTII	YQDIALATR	525.8	795.43
NPTII IS	YQDIAL*ATR	529.3	766.43
GAT	HAEEILR	434.2	730.3
GAT IS	HAEEIL*R	437.7	737.3
PMI	VQNAAGDIVSLR	621.8	759.4
PMI IS	VQNAAGDIVSL*R	625.3	766.4
